# The Drosophila BTB Domain Protein Jim Lovell Has Roles in Multiple Larval and Adult Behaviors

**DOI:** 10.1371/journal.pone.0061270

**Published:** 2013-04-19

**Authors:** Sonia M. Bjorum, Rebecca A. Simonette, Raul Alanis, Jennifer E. Wang, Benjamin M. Lewis, Michael H. Trejo, Keith A. Hanson, Kathleen M. Beckingham

**Affiliations:** Department of Biochemistry and Cell Biology, Rice University, Houston, Texas, United States of America; VIB & Katholieke Universiteit Leuven, Belgium

## Abstract

Innate behaviors have their origins in the specification of neural fates during development. Within Drosophila, BTB (**B**ric-a-brac,**T**ramtrack, **B**road) domain proteins such as Fruitless are known to play key roles in the neural differentiation underlying such responses. We previously identified a gene, which we have termed *jim lovell* (*lov*), encoding a BTB protein with a role in gravity responses. To understand more fully the behavioral roles of this gene we have investigated its function through several approaches. Transcript and protein expression patterns have been examined and behavioral phenotypes of new *lov* mutations have been characterized. Lov is a nuclear protein, suggesting a role as a transcriptional regulator, as for other BTB proteins. In late embryogenesis, Lov is expressed in many CNS and PNS neurons. An examination of the PNS expression indicates that *lov* functions in the late specification of several classes of sensory neurons. In particular, only two of the five abdominal lateral chordotonal neurons express Lov, predicting functional variation within this highly similar group. Surprisingly, Lov is also expressed very early in embryogenesis in ways that suggests roles in morphogenetic movements, amnioserosa function and head neurogenesis. The phenotypes of two new *lov* mutations that delete adjacent non-coding DNA regions are strikingly different suggesting removal of different regulatory elements. In *lov^47^*, Lov expression is lost in many embryonic neurons including the two lateral chordotonal neurons. *lov^47^* mutant larvae show feeding and locomotor defects including spontaneous backward movement. Adult *lov^47^* males perform aberrant courtship behavior distinguished by courtship displays that are not directed at the female. *lov^47^* adults also show more defective negative gravitaxis than the previously isolated *lov^91Y^* mutant. In contrast, *lov^66^* produces largely normal behavior but severe female sterility associated with ectopic *lov* expression in the ovary. We propose a negative regulatory role for the DNA deleted in *lov^66^*.

## Introduction

Understanding the molecular origins of behavioral responses is a central goal of neuroscience. Innate behaviors are a particular focus since these reflexive activities originate in neural circuitry “hard wired” into the organism during development to give robust, invariant responses. As the most intensely studied complex multi-cellular organism, Drosophila offers the best possibilities for understanding the processes by which neural circuitry is laid down in development.

Within Drosophila, the relatively simple larval nervous system, which is formed during embryogenesis, is providing many insights into neurogenesis and neural differentiation. The critical role of transcription factor cascades has emerged from studies of the peripheral nervous system (PNS), where major components of the transcriptional hierarchies that give rise to various classes of sensory neurons have been identified (reviewed in [1], [2]). Similarly, gene profiling of the CNS midline cells has identified cell-type specific signatures of transcription factor expression [3], [4], whose roles in neural specification is being confirmed by genetic studies [5], [6]. However, many further steps in the processes leading to final differentiation of individual neurons remain to be identified.

Through a screen for defective responses to gravity, we previously identified CG16778 as a neurally expressed gene that influences behavioral responses [7]. We have named the gene *jim lovell* (*lov*) in honor of this astronaut’s pioneering work in microgravity. *lov* encodes a putative transcription factor of the BTB/POZ domain family. These proteins contain a ∼120 amino acid domain, initially identified in the Drosophila proteins **B**ric-a-brac, **T**ramtrack and **B**road [8] and in **Po**xvirus **z**inc finger proteins [9], that acts as a protein-protein interaction module mediating homo- and hetero-dimer formation and sometimes higher order oligomerization in various family members. Lov belongs to the more closely related tramtrack (ttk) subgroup [10] that has a BTB/POZ domain with three distinct regions of high conservation [11]. Fruitless, the master regulator for male courtship behavior, is also a ttk subgroup member [12]. Most of the Drosophila ttk proteins are known/putative transcription factors containing a zinc finger DNA binding domain. However, Lov belongs to a smaller subset consisting of Pipsqueak, Ribbon, Bric-a-brac, Pielke, and three uncharacterized proteins, that all contain a modified helix-turn-helix DNA binding domain termed the pipsqueak domain [11], [13]. These conserved domains thus suggest roles for *lov* in transcription regulation mediated via protein-protein interactions. Based on a partial cDNA sequence for CG16778, the gene was initially proposed to belong to the tyrosine kinase family and was named Tyrosine-kinase related (Tkr) [14]. Given that the protein does not function as a kinase, the authors of that previous study have agreed to the name change we propose here (H. Jackle, personal communication). CG16778 was also identified as gene BTB III in a study aimed at identifying BTB-containing genes in Drosophila [15].

We present here an investigation of *lov* function based on analysis of its expression and on study of new *lov* mutants derived from our initial gravitactic mutant, *lov^91Y^*. These studies show that *lov* is widely expressed in the embryonic nervous system and indicate a role in the late development of some sensory neurons, potentially producing distinctions in the properties of closely related neurons within a given class. We also demonstrate that *lov* is essential for proper execution of several innate behaviors - ranging from coordinated locomotor responses in larvae to negative gravitactic climbing and male courtship behavior in adults. We further provide evidence that *lov* has non-neural roles in early embryonic pattern formation and in the testis.

## Materials and Methods

### Mutation Generation by Imprecise Excision

Deletion mutations *lov^38^*, *lov^47^* and *lov^66^* were generated by mobilizing the *w^+^ P{GawB*} [16] insertion of *lov^91Y^* [7] with the Δ2–3 transposase at 99B [17]. Standard genetic crosses were used to generate lines of *w^−^* viable mutations. Each deletion was initially identified by genomic PCR and the precise endpoints for each deletion were determined by sequencing appropriate PCR fragments.

### Fly Stocks and Genetics

Deficiency *SB1* (also known as *Df(2R)Kr10*), which removes the entire *lov* locus [18], was obtained from the Bloomington Drosophila Stock Center (BDSC) in stock 4961. For behavioral analysis, mutations *lov^38^*, *lov^47^*, *lov^66^*, *lov^91Y^* and deficiency *SB1* were all isogenized by six rounds of outcrossing to a *w^+^*; *Sco/CyO* stock. *SB1* and *lov^66^*, which cannot be maintained as homozygous stocks, are carried over the *CyO* chromosome from this *w^+^; Sco/CyO stock*. Stock 5702 (*w^−^*; *Sco/CyO-GFP*) from BDSC was used to generate *lov^47^/CyO-GFP* and *lov^66^*/*CyO-GFP* lines for larval and embryo analysis, respectively. For ectopic expression of *lov*, the following GAL4 drivers were obtained from BDSC: *(GAL4::VP16-nos.UTR) CG6325^MVD1^* (stock 4937), *P{GawB*}*c204* (stock 3751), *P{GawB*}*c355* (stock 3750) and *P{GawB*}*c532* (stock 30841).

### Semi-quantitative RT-PCR

RNA was prepared from various tissues or life cycle stages using Trizol lysis as described in [19]. cDNA was prepared using Superscript III reverse transcriptase (Invitrogen) and a random hexamer mix (New England Biolabs). For detection of processed *lov* transcripts, the primer 5′ TCAACTTCTGGTGGGCGTCCTTTA 3′, which is the reverse complement of residues 41–64 of the first common exon of the four *lov* transcripts, was paired with primers specific for the immediate upstream 5′UTR exon of each transcript (see below). PCR (30 cycles) at appropriate annealing temperatures was then used to generate fragments specific for each *lov* mRNA. Primers for transcripts from the ubiquitously expressed Actin gene at 57B were used to standardize output for analysis. Fragments were detected after agarose gel electrophoresis and ethidium bromide staining. Primer sequences were as follows.

Transcript A - 5′ ATCCGAGTGTCATCTTCAACGCGA 3′.

Transcript B - 5′ ACATACGCTCATTCGTTACCCGCT 3′.

Transcript C - 5′ TGTCTTGAACAGAACTATATTGTG 3′.

Transcript D - 5′ GTTTCCAAAGAAGCAATCAAACGGC 3′.

Actin 57B forward –5′ TTCCAAGCCGTACACACCGTAACT 3′.

Actin 57B reverse –5′ TCATCACCGACGTACGAGTCCTTCT 3′.

### Antibody Generation

**Figure 1:**
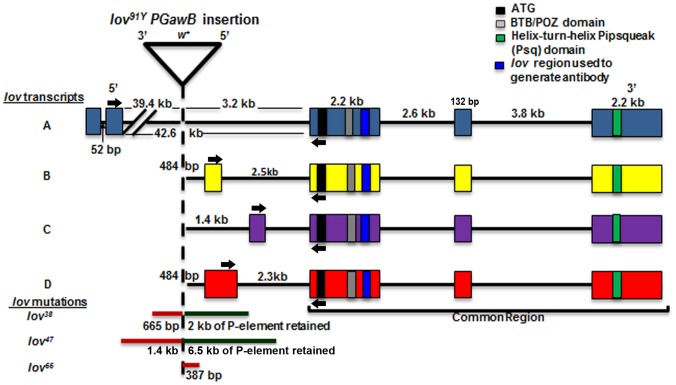
Transcripts and mutations of the Drosophila *jim lovell* locus. The exon structure of the four *lov* transcripts is shown. The three common exons encode the same BTB/POZ domain protein. Structural motifs of the protein and the region used to prepare antibodies are indicated. Arrows indicate the positions of the primers used to probe for expression of the four transcripts. The original *lov^91Y^* P{GawB} insertion and three deletion mutations (*lov^38^*, *lov^47^* and *lov^66^*) generated by imprecise excision of the transposon are shown.

A 218 amino acid region of the *lov* coding sequence with no detectable similarity to any other protein sequence in the Drosophila genome was chosen for antibody generation. This region lies within the first protein coding exon (common to all four transcripts), 948 residues downstream of the ATG start site and 270 residues downstream of the BTB domain (Figure 1). Primers (5′ CTGGAAGTTCTGTTCCAGGGGCCCCTGGGATCC 3′ and 5′ GAATTCCCGGGTC GACTCGAGCGGCCGCAT 3′) that add an upstream *BamH I* site and downstream *EcoR I* site were used to generate the required PCR fragment from *lov* cDNA clone GH08221 (Berkeley Drosophila Genome Project) for CG16778. After initial cloning into pCR4-TOPO (Invitrogen), the coding region was transferred to expression vector pGEX-6P-1 as a *BamH* I/*EcoR* I fragment, to generate a GST-*lov* fused coding sequence. After induction, fusion protein was collected on a glutathione column. The Lov region was released by cleavage with PreScission Protease (GE Life Sciences) and used to raise antibodies in a rabbit and a guinea pig (Cocalico Biologicals, Inc). The recombinant Lov protein fragment cross-linked to NHS-activated Sepharose resin (GE Life Sciences) was used for affinity purification.

### UAS-*lov* Construct

The entire *lov* protein coding sequence in cDNA clone GH08221 was amplified using primers (5′ GTTGAATTCCTGGATACGAGAATTGAAGCACGC 3′ and 5′ GTTAGATCTGTTTCAATCATGCCCGGTC 3′) that add an upstream *EcoR I* site and a downstream *Xho I* site. The resulting PCR fragment was cloned into these two sites in the pUAST vector [16] using standard cloning techniques. The Lov protein sequence within the final construct was confirmed by DNA sequencing. Transgenic animals were generated by GenetiVision (Houston) and homozygous viable lines carrying single copies of the construct on each of the major chromosomes were prepared by standard genetic procedures. A line carrying UAS-*lov* on the second chromosome was used here.

### Embryo Immunostaining

Standard procedures were used to collect embryos and then to dechorionate and fix them in 3% paraformaldehyde and to remove their vitelline membranes. Embryos were stored at −20°C in methanol prior to staining. After rehydration in phosphate buffered saline (PBS), embryos were blocked with 5% goat serum in PBS and then stained with primary antibodies. Antibodies used were as follows: guinea pig anti-Lov 1∶500, rabbit anti-Lov 1∶200, mouse 22C10 (Developmental Studies Hybridoma Bank) 1∶200. For single labeling, biotin-labeled secondary antibodies (Vector Labs, 1∶500 dilution) were used, followed by streptavidin-horse radish peroxidase (Pierce) and detection via metal-enhanced 3,3′-Diaminobenzidine (DAB) treatment (Pierce). For double labeling with anti-Lov and 22C10, alkaline phosphatase (AP) labeled anti-mouse secondary antibodies (Promega, 1∶500 dilution) were also applied and detected with an X-phosphate nitroblue tetrazolium detection kit (Vector labs). For examination of *lov^66^*/*lov^66^* embryos, a *lov^66^*/*CyO-GFP* stock was used. Embryos were co-stained with anti-GFP peptide antibody (Clontech Laboratories, Inc, 1∶500 dilution) and anti-Lov antibody to identify *lov^66^* homozygous embryos.

### Hatch Rate Determinations and Egg Shell Analysis

Males and virgin females 3–6 days of age were set up in egg lay dishes over small Petri dishes containing grape juice agar smeared with yeast paste. 12–15 male-female pairs were used per egg lay dish. After discarding the initial plate, 100 eggs were collected from each of three successive overnight collections. The eggs were arrayed in a 10×10 grid on a new plate and after 36 hours, the number of unhatched eggs was counted. For each male/female combination tested, at least two separate sets of mating pairs were set up to give at least six estimates of egg hatch rate. For eggshell analysis, after preparing a 10×10 grid of eggs, eggshells and dorsal appendages were scored and classified as described in the text.

### Larval Locomotion and Food Shoveling Assays

Assays were modified from the protocols described by Neckameyer [20]. Four-hour egg lays were collected using egg lay dishes as described above. For locomotor assays, 24 hours later, batches of 30 newly hatched larvae were transferred to grape plates covered with 2.0 grams of yeast paste (40% w/v dry yeast powder). 72 (+/−2) hours after egg laying, larvae were washed free of food using fine Nitex meshes and transferred in batches onto 2% agar plates in preparation for assays. Individual larvae were then placed on a fresh 2% agar plate, allowed one minute to acclimatize and then the number of locomotor contraction waves (strides) in one minute was counted. In all genotypes but *lov^47^*, only forward strides were seen. But in *lov^47^*, trains of backward strides were also detected. To quantitate this effect, any larva showing three or more consecutive backward locomotor strides was scored as showing spontaneous backward movement. For food shoveling assays, newly hatched first instar larvae were collected from egg lay plates, and placed on 2% agar plates for one hour prior to assay. Individual larvae were then transferred to a 2% agar plate coated with a layer of 2% w/v yeast paste. After a minute of acclimatization, the number of mouth hook contractions (shovels) in one minute was scored. *lov^66^* homozygous larvae were collected from the *lov^66^*/*CyO-GFP* stock based on lack of GFP fluorescence. At least 100 larvae of each genotype were tested in each assay.

### Adult Climb Tests

Newly eclosed males were placed in individual food vials and aged for two days. Each was then transferred to an empty food vial with a circumferential marking line 5 cm from the base of the vial. The fly was then tapped gently to the bottom of the vial and the time to climb to the 5 cm mark was recorded. Each fly was tested 10 times with a one-minute rest period between tests. Any trial in which a fly failed to reach the 5 cm line in one minute was scored as a climb failure. At least 50 adults of each genotype were tested.

### Male Courtship and Activity Assays

Courtship assays were modified from the protocol described previously [7]. Newly eclosed naïve males were collected and aged individually in food vials for 7 days before testing with single virgin 3–5 day old Canton-S control females. Wheel-type courtship chambers were used [7]. Flies were transferred to the chambers without anesthetization. Behavior was video-recorded and the amount of time each male spent performing any element of the courtship ritual (orienting to the female, tapping, wing extension, licking, and attempting copulation) in a 10 minute interval was determined. Two Courtship Indices (C.I.s) were calculated. The “directed” C.I. was calculated as the fraction of total time spent performing these rituals towards the female. The “non-directed” C.I. was calculated as the fraction of time preforming courtship elements not focused on the female. At least 10 males were tested for each genotype. General locomotor activity was determined for males alone in the courtship chambers by positioning a black line under the chamber at its diameter and determining how many times the male crossed the line in a 10 minute period. At least 25 males were tested for each genotype.

## Results

### The Multiple Transcripts of *lov* Show Tissue and Developmental Stage Specific Expression

**Figure 2:**
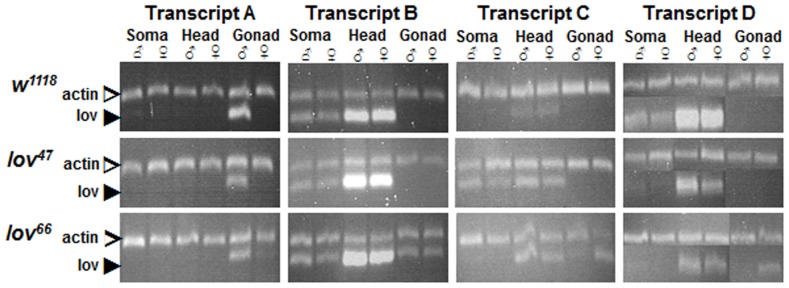
Tissue specific expression of the *lov* transcripts in adults. Semi-Q RT-PCR was used to detect *lov* transcripts in the bodies (soma), heads and gonads of male and female adults from a control (*w^1118^*) strain and *lov^47^* and *lov^66^* mutant lines. Primers as indicated in Figure 1. In all cases, the fragments amplified span intron-exon boundaries to limit detection to mature transcripts. A fragment from the ubiquitous Actin mRNA (Actin at 57B) was amplified in parallel as the control. At least two separate RNA preparations for each tissue were used for transcript quantitation. The PCR fragment for transcript D was very close in size to the Actin PCR fragment. To quantitate this transcript, aliquots of separate transcript D and Actin PCR reactions for each RNA sample were run in parallel in separate gel lanes.

**Figure 3:**
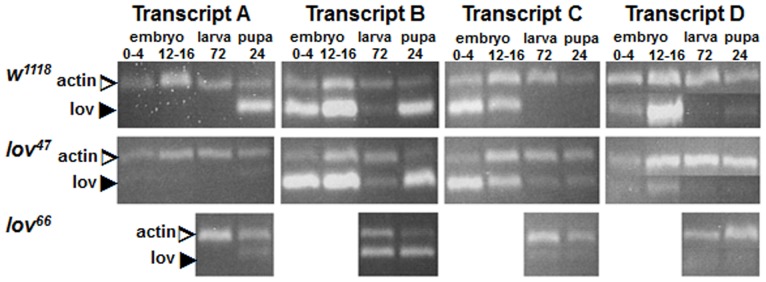
Developmental expression of the *lov* transcripts. Semi-Q RT-PCR, as described for Figure 2, was used to detect *lov* transcripts in embryos 0–4 and 12–16 hours after egg laying (AEL), larvae 72 hours AEL, and pupae 24 hours after pupation from control (*w^1118^*) and *lov^47^* and *lov^66^* mutant flies. *lov^66^* is maintained as a balanced (*lov^66^/CyO*) stock (see text) and transcripts for homozygous *lov^66^* embryos could not be reliably evaluated. At least two separate RNA preparations for each stage were used for transcript quantitation.

The structure of the *lov* locus as predicted by Flybase in 2008 is shown in Figure 1. The four *lov* transcripts all generate the same protein encoded by three common 3′ exons of the gene. However, three different promoters are used and although transcripts B and D share a promoter, transcript D contains 5′ UTR sequences not present in transcript B. Thus unique 5′ non-coding sequences are present in each of the mRNAs. The transcript B/D promoter and C promoters are close to the protein coding exons, whereas the promoter for transcript A is remote, lying ∼ 43 kb upstream of the first protein coding exon. We used Semi-quantitative (Semi-Q) RT-PCR to investigate the expression patterns of the transcripts. A primer from the first protein coding exon was paired with transcript-specific primers derived from the non-coding 5′exons of the individual mRNAs so as to generate PCR fragments for each of the final four mRNAs. Figures 2 and 3 show the tissue distribution of the transcripts in adults and their developmental expression patterns, respectively.

The various transcripts show strikingly different expression profiles. Transcript A is testis-specific and transcript C is almost entirely limited in expression to the early stages of embryogenesis. Transcripts B and D, which share a transcription start site, show considerable overlap in their expression patterns. Both are more strongly expressed in the adult head rather than the soma, suggesting that they have neural roles. However both are also expressed in the early embryo along with transcript C indicating non-neural functions in early development, as described below. Transcript D appears to be the dominant neural transcript since it shows a greater differential presence in adult head versus soma than transcript B. Further, it shows much stronger expression in late embryos, during the neural developmental stages, than transcript B. As described below, the late embryonic expression of Lov is entirely neural. During the larval stages, very little *lov* transcription is detected and Lov protein expression in the nervous system fades away. But as the remodeling of the pupal stages begins, transcript B expression is again expressed (Figure 3) and Lov protein becomes detectable within proliferating CNS neurons (data not shown).

During the course of our work, transcript A, which was originally part of the Flybase description of the gene, was removed by the Flybase curators. However our Semi-Q RT-PCR experiments, in which we probed for processed mRNAs from the locus, establish that transcript A is indeed expressed in the organism as a testis-specific transcript (Figure 2). This transcript is first detected in early pupal stages as the male gonad begins its final development (Figure 3). Recent work indicates that *lov* transcription in the testis is regulated by *wake-up-call*, a *lin-52* paralog [21].

### Embryonic Lov Protein Expression Suggests Distinct Roles in Early Pattern Formation and in Neural Differentiation

To examine Lov protein expression we generated polyclonal antibodies against a unique protein-coding region (Figure 1) in rabbit and guinea pig and subjected them to affinity purification. We were able to confirm the specificity of these antibodies for protein immunolocalization in three separate ways. First, as predicted, Lov immunostaining is nuclear and the nuclear patterns correspond well to cytoplasmic *in situ* hybridization patterns for *lov* mRNA probes described previously [14], [15]. Second the antibodies stain every neural nucleus after ectopic expression of Lov protein throughout the embryonic nervous system using the *elav* GAL4 driver and a UAST-*lov* construct [22]. Finally, one of our *lov* mutations (*lov^47^*) results in loss of elements of the antibody immunostaining from components of the nervous system (see below).

**Figure 4:**
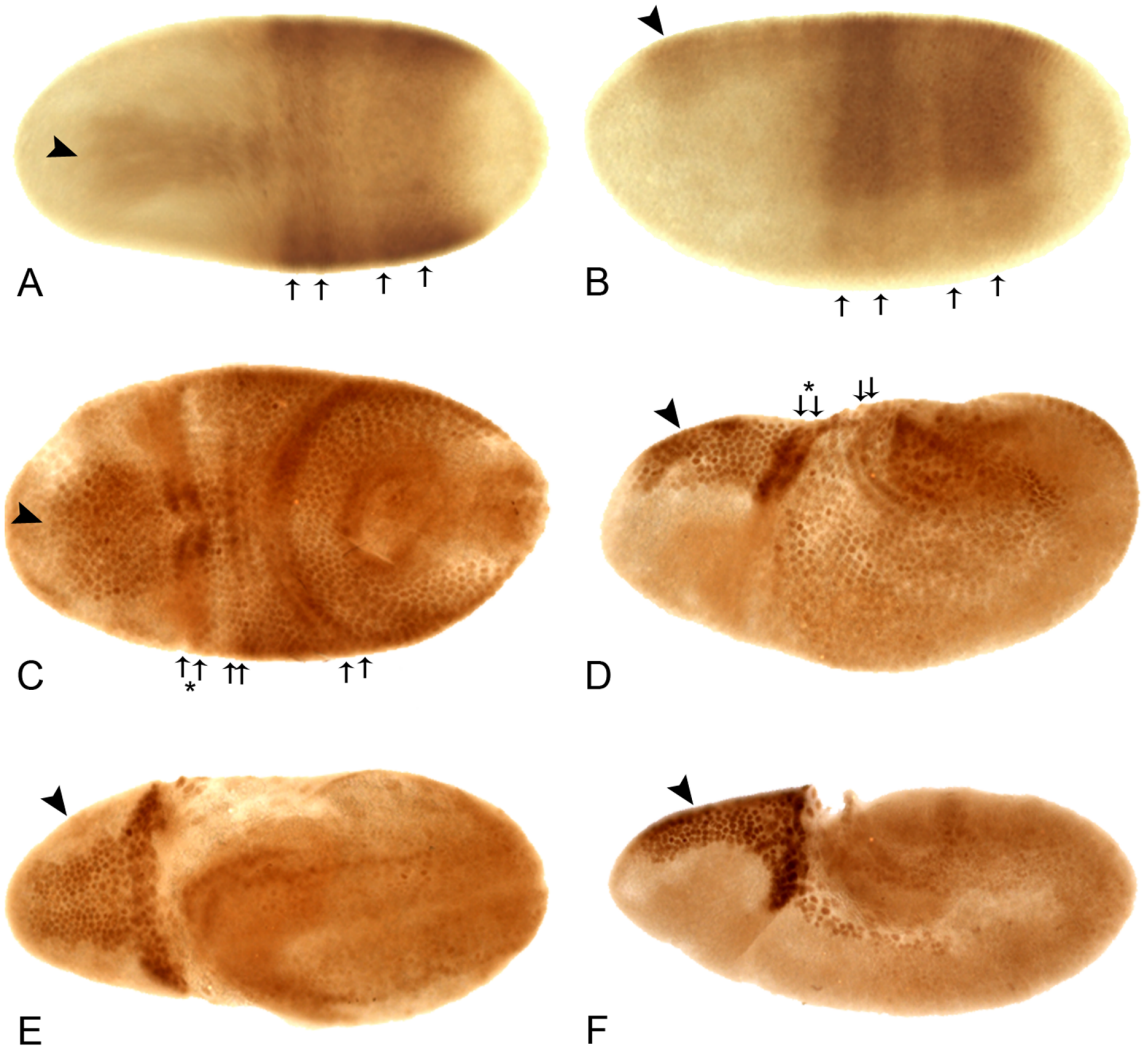
The early embryonic expression pattern of Lov protein. All embryos are anterior to left. **A**, **C**, and **E** - views of the dorsal surface; **B**, **D**, and **F** - lateral views, dorsal uppermost. At cellular blastoderm (**A**, **B**) Lov staining appears across the dorsal midline as an anterior “wedge” (arrowhead) and a “saddle” of two pairs of diffuse stripes (arrows) at ∼10–50% egg length. In early germ band extension (**C**, **D**), staining in the wedge intensifies (arrowhead) and the diffuse saddle stripes (arrows) sharpen into paired rows of nuclei spanning the positions of ectodermal folds (see text). A further pair of rows of nuclei staining for Lov across the dorsal midline develops at the edges of the cephalic furrow (arrows and asterisk). The staining in these rows is particularly intense where they traverse the wedge of Lov stain. In late germ band extension (**E**, **F**), the wedge staining (arrowheads) can be seen to lie within the procephalic neuroectoderm. Intense staining is also seen in the polyploid nuclei of the amnioserosa at its junction with the cephalic furrow and in its lateral longitudinal extensions. In **E**, paired rows of Lov staining nuclei are also seen at the periphery of the extending germ band.

We have focused on the pattern of Lov protein expression during embryogenesis. Our RT-PCR experiments reveal a pronounced shift in *lov* mRNA expression, from transcript C to transcripts B and D, as embryogenesis progresses (see above). These changes are associated with two distinct phases of Lov expression: an early non-neural phase and a later phase associated with differentiation of the CNS and PNS. The early pattern is shown in Figure 4. The newly laid egg contains no Lov protein, in agreement with the absence of *lov* mRNA in the ovary (Figure 2). At cellular blastoderm, Lov appears in two distinct regions. A “wedge” of Lov protein spanning the anterior dorsal midline appears simultaneously with a broad “saddle” of expression, also across the dorsal midline, that stretches from ∼10–50% along the egg length and around ∼60% of the embryonic circumference (Figure 4 A, B). This saddle contains two prominent dark stripes at its anterior edge separated from a second pair of more diffuse dark stripes, of which the most posterior is significantly broader. The narrow pointed region of the wedge of Lov expression can also be seen to contain two or three darker stripes.

As germ band extension begins, the diffuse stripes within the wedge and saddle sharpen into discrete pairs of rows of cells with darkly staining nuclei (Figure 4 C, D). These rows correspond to the opposing edges of the folds in the dorsal ectoderm generated by germ band extension: these folds are the cephalic furrow, the anterior and posterior transverse ectodermal folds and the amnioproctodeal invagination. The staining in the pair of rows at the edge of the cephalic furrow is particularly intense. Cells at the edges of the embryonic folds may have special properties such as enhanced rigidity and Lov may in someway contribute to these characteristics. Lov expression in the remainder of the wedge also intensifies and becomes recognizable as lying within the procephalic neuroectoderm (pne) region, the source of many brain neurons. At full germ band extension, Lov is expressed in the dorsolateral ectoderm and in another pair of highly staining rows of cells around the periphery of the extended germ band (Figure 4 E). These cells are again positioned at the edges of dynamic folds and may also need mechanical reinforcement. Finally strong staining appears in the developing amnioserosa. This structure, which forms from cells between the tip of the extended germ band and the dorsal region of the cephalic furrow has roles in dorsal closure and germ band movement. The strongest staining in the embryo at full germ band extension is in the pne (Figure 4 F), where presumptive proneural cells are closely apposed to a band of strongly staining polyploid amnioserosal cells lining the cephalic furrow.

**Figure 5:**
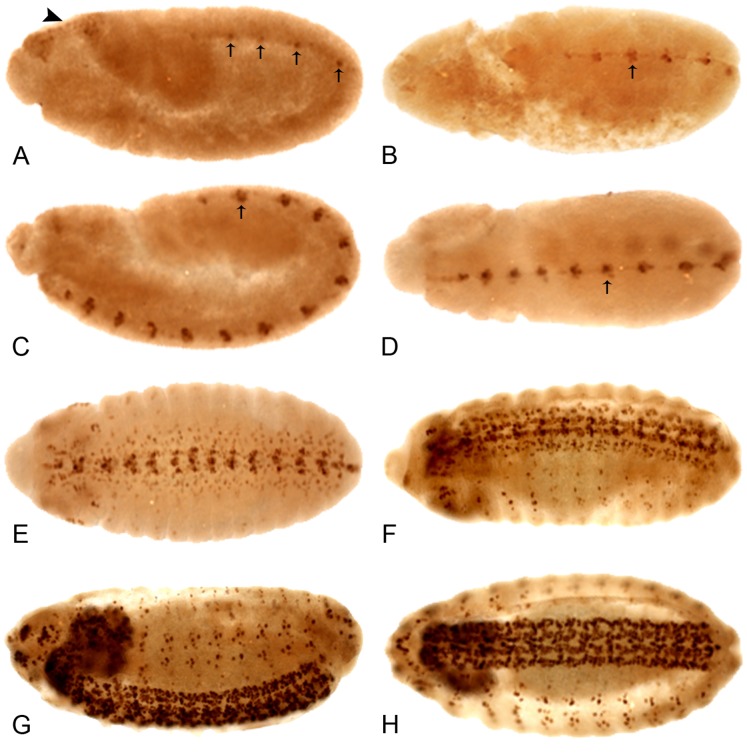
The late embryonic expression pattern of Lov protein. All embryos are anterior to the left. **A**, **C**, and **G** - lateral views, dorsal uppermost; **B**, **D**, **E**–**H** - dorsal views. At the end of germ band extension (**A**) the early Lov pattern (Figure 4) disappears and a single nucleus at the midline of each parasegment begins to express Lov. As germ band retraction proceeds (**B**–**D**), more nuclei at the developing CNS midline express Lov. Nuclei in the lateral regions of the CNS (**E**) the brain lobes (**E**, **F**) and along the longitudinal connectives (**F**) develop staining as germ band retraction is completed and dorsal closure begins. PNS staining is first detected in early dorsal closure (**F**). At ∼ stage 15, CNS and PNS staining are maximal (**G**, **H**) and CNS staining has a mesh-like appearance (**H**).

After full germ band extension, Lov staining disappears and the embryo is briefly devoid of Lov expression. But as segregation of neuroblasts from the ectoderm begins, a second phase of expression is initiated. A single nucleus in the midline of each developing thoracic and abdominal segment begins Lov expression (Figure 5 A). Further Lov positive CNS nuclei both at the midline and more laterally in the ventral nerve cord (VNC) appear during germ band retraction (Figure 5 B–D) so that a pattern dominated at first by nuclei at the positions of the longitudinal connectives is formed followed by a pattern with two additional longitudinal strong bands of nuclei at the lateral edges of the VNC. Lov-positive nuclei connecting these bands also develop such that the late Lov CNS staining has a mesh-like appearance (Figure 5 D–F). Using *in situ* hybridization, the Crews lab has previously characterized *lov* mRNA expression in CNS midline cells and other components of the CNS and PNS at later stages [3], [4]. Our Lov immunolocalizations match in nuclear protein staining the cytoplasmic *lov* transcript patterns detected in the Crews lab.

**Figure 6:**
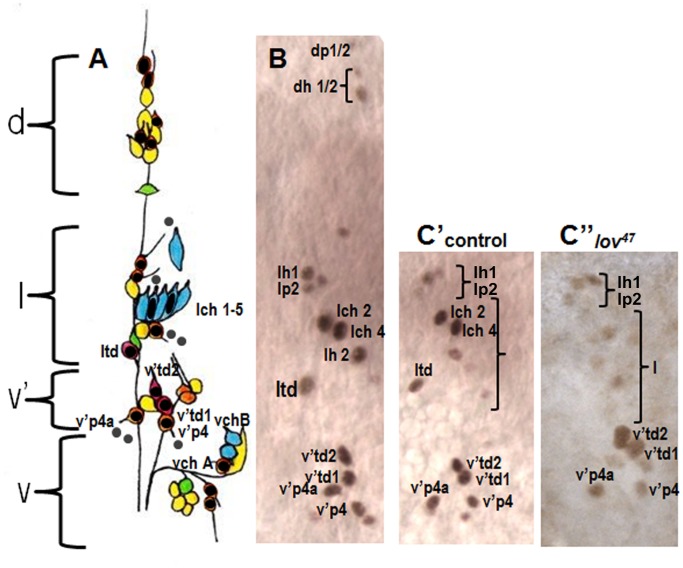
Lov expression in the embryonic abdominal PNS. **A** - a cartoon of the four clusters (dorsal (d), lateral (l), ventral’ (v′) and ventral (v)) of sensory neurons within each hemisegment of the abdominal segments (redrawn from [2]). Neurons are color coded according to class as follows: orange - external sense organ, pink - tracheal dendrite, blue - chordotonal, yellow - dendritic arborization, green - bipolar dendritic. Key neurons discussed in the text are labeled. Neuronal nuclei expressing Lov (shown by black dots or ovals) were identified in embryos co-stained with anti-Lov and 22C10. Grey dots = putative non-neuronal support cell nuclei that express low levels of Lov. Nomenclature for all neurons as in [24] except that the neurons of the lateral chordotonal organ are labeled lch1-5. **B** - Actual example of wild type Lov-staining nuclei at stage 15 in clusters d, l, and v′ of a single abdominal hemisegment. **C** - anti-Lov (brown) and 22C10 (blue) costaining of the lateral chordotonal organ in a control embryo to demonstrate Lov limitation to lch2 and lch4. **D** - 22C10 staining of a *lov^47^* homozygous embryo demonstrating the presence of all five chordotonal neurons in the lateral chordotonal organ. **E**′, **E**′′ - loss of Lov staining in chordotonal organ neurons lch2 and 4 of a *lov^47^* mutant embryo. **E**′ shows control staining in clusters l and v′. **E**′′ shows staining in equivalent regions of a *lov^47^* embryo. Loss of staining in td neuron ltd is also seen in this *lov^47^* embryo.

Although great progress in identifying the neuronal sub-types within the embryonic CNS has been made [23], at this point the PNS of the embryonic abdominal segments is better characterized, with four clusters of neurons (dorsal, lateral, ventral and ventral’) showing a highly stereotypical pattern in each hemisegment [24]. We therefore focused on analyzing Lov expression in these segments as a means of gaining insight into Lov’s roles in neural development. PNS Lov staining is not detected until after germ band retraction and initiation of dorsal closure, that is, as the final neurons of the PNS are developing and beginning terminal differentiation. A single darkly staining nucleus first appears in the lateral cluster, rapidly followed by groups of nuclei in all four clusters. The number of Lov-positive nuclei and the overall Lov staining intensity increases as dorsal closure proceeds such that stage 15 represents a point of maximal expression. We used co-staining with antibody 22C10, which stains all sensory neurons [25], to identify the PNS nuclei expressing Lov at this stage. Almost all stained nuclei are neuronal although faint staining was detected in some non-neuronal cells. From their positions we deduce that these are support cells in the external sense organ neural lineages. Figure 6 A shows a diagram of the sensory neurons in a typical abdominal hemisegment, indicating all nuclei identified as Lov-positive. Figure 6 B shows the actual ∼stage 15 Lov staining pattern in a single hemisegment for the dorsal, lateral and ventral’ clusters.

Five classes of sensory neurons are present in the abdominal PNS; external sense organ (eso) neurons, chordotonal (ch) neurons and three classes of multiple dendritic neurons – the dendritic arborization (da), bipolar dendritic (bp) and tracheal dendrite (td) classes. With the possible exception of one da class cell (Figure 6 A), Lov is not expressed in any neurons of the bp or da classes. All three td neurons show strong nuclear staining and almost all eso class nuclei are also Lov positive, although some stain quite weakly and no staining was ever detected in two eso neurons of the ventral’ cluster. Strikingly, of the eight chordotonal organ neurons present per hemisegment, six showed no Lov expression, but two express Lov strongly. These are neurons 2 and 4 of the lateral pentascolopale organ (lch 2 and 4 where lch 1 is the nearest neuron to the intersegmental nerve) (Figure 6 C). The nucleus of neuron 2 of this cluster is the first peripheral neuron to begin expressing Lov (see above). Lov expression is thus not limited to any particular class of PNS neurons and is not expressed in all members of the classes in which it is expressed.

At stage 15, the nuclei of lch2 and 4 together with the two td neurons of the ventral’ cluster show the strongest Lov staining. In the final stages of embryogenesis and immediately before cuticle formation, these four nuclei are often the only peripheral neurons retaining Lov expression, creating a distinctive pattern of four dots in each abdominal segment.

### Phenotypes Associated with Deletion Mutations of the *lov* Locus

We identified a single mutation to the *lov* locus (*lov^91Y^*) as part of a screen for mutations that affect gravity responses in Drosophila [7]. The *P{GawB}* element associated with *lov^91Y^* is inserted close to the transcription start sites for the B, C and D *lov* mRNAs and within the large intron of the A transcript. Imprecise excision of the *lov^91Y^* transposon generated three mutations that delete genomic DNA from the locus (Figure 1). *lov^38^* and *lov^47^* delete sequences upstream of the *lov^91Y^* insertion point, with the *lov^38^* deletion lying entirely within the 1.4 kb *lov^47^* deletion. In contrast, *lov^66^* deletes ∼400 bp of DNA downstream of the *lov^91Y^* transposon. The mutations thus delete intronic or flanking DNA not present in any final transcripts, with *lov^38^* and *lov^47^* deleting overlapping DNA that is distinct from that of the *lov^66^* deletion.

All three of the new deletion mutations produce viable adults with normal external morphology suggesting that any phenotypic defects are limited to behavior and/or reproductive processes rather than developmental events. As a consequence of their origins as imprecise excisions of the *w^+^ P{GawB}* transposon of *lov^91Y^*, these new mutations were initially in a *w^−^* background. To allow comparisons to *lov^91Y^* and avoid possible effects of the *w* ^−^ background on adult behavior [26], the new mutations and *lov^91Y^* were all subjected to serial outcrossing to put them into the same *w^+^* background. All of the studies described here were performed with mutant lines in this genetic background.

#### Loss of *lov* expression in *lov^47^* leads to defects in larval locomotion and feeding and adult locomotion and courtship

**Figure 7:**
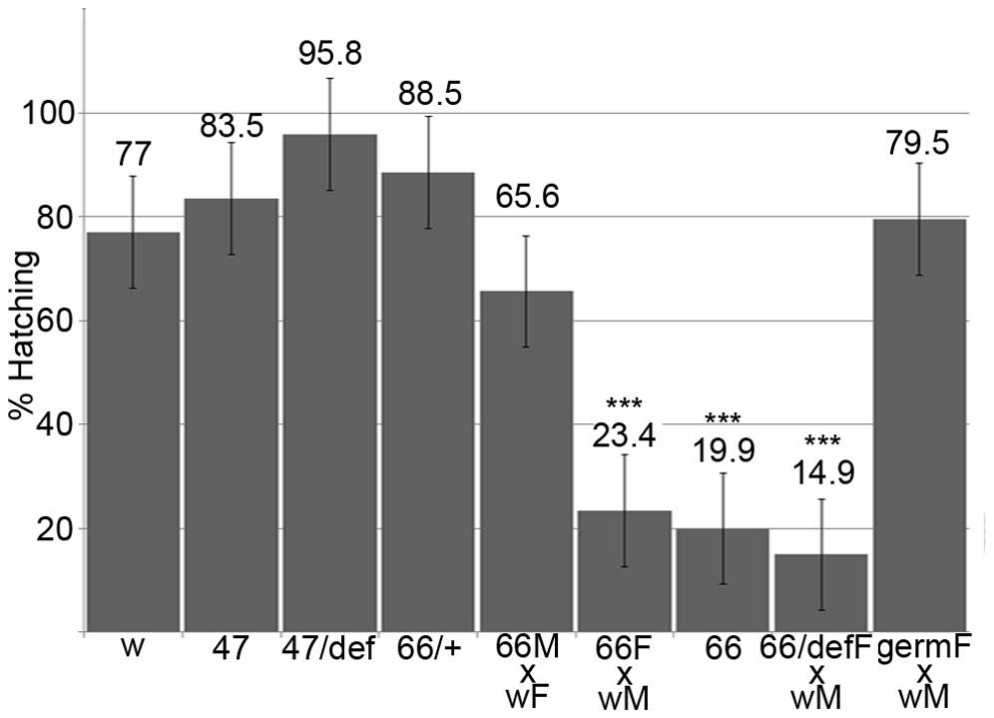
Hatch rates for eggs from *lov^47^*, *lov^66^* and related genotypes. Batches of eggs from stocks or crosses of the genotypes indicated were collected and scored for hatching as described in Material and Methods. w = *w^1118^*, 47 = *lov^47^*, 66 = *lov^66^*, def = *lov* deficiency chromosome *SB1,*+* = CyO* balancer, germ = UAS-*lov* in the ovarian germline under the GAL4 driver P (GAL4::VP16-*nos*.UTR) CG6325^MVD1^. M = male, F = female. Hatch rates for the *lov^66^*/*CyO* stock and the *lov^47^*/*def* stock were adjusted to correct for death of *CyO*/*CyO* and *def*/*def* homozygotes. At least six separate collections, totaling at least 600 eggs, were scored for each genotype. One way Anova and a Dunnett’s test were used to determine statistical significance *** = p<0.001 compared to *w^1118^.*

**Figure 8:**
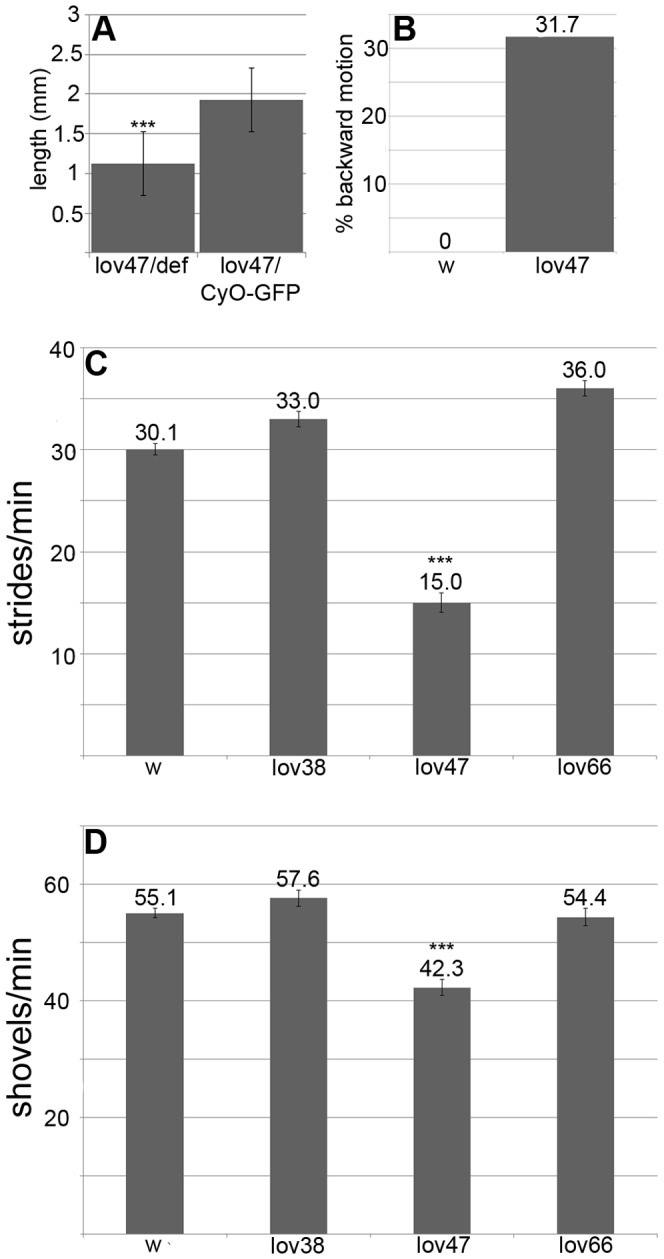
Growth and behavioral defects in *lov^47^* larvae. **A** - poor growth of *lov^47^* larvae in rich medium. *lov^47^* hemizygous (lov47/def) and heterozygous (lov47/CyO-GFP) larvae from a *lov^47^*/*CyO-GFP* stock grown on yeast paste were sorted based on GFP fluorescence at 87 hours AEL and imaged under a dissecting microscope with a length gauge, after brief ether anesthetization. **B** - percentage of larvae at 72 hours AEL showing backward locomotion (as defined in Material and Methods) for *lov^47^* and *w^1118^* (w). **C** - forward locomotion rates at 72 hours AEL for *w^1118^*, *lov^38^*, *lov^47^*, and *lov^66^* homozygous larvae. **D** - food shoveling rates at 72 hours AEL for larval genotypes as in **C**. Statistics as for Figure 7. *** = significantly decreased (p<0.001) as compared to *w^1118^*.

Although *lov^47^* homozygous and hemizygous females are highly fertile in terms of eggs laid and egg hatch rate (Figure 7), the resulting larvae show delayed development and poor growth. Fewer individuals survive to adulthood and surviving adults are often reduced in size. Even when fed on a diet of pure yeast paste, *lov^47^* larvae are significantly smaller than Canton-S controls (Figure 8 A). We detected two behavioral defects in the larvae that could contribute to this poor growth. From hatching onwards *lov^47^* larvae are sluggish, showing less than half the locomotor activity of controls (Figure 8 C). Locomotion is also often deregulated. Whereas wild type larvae only show reflexive backward movements in response to contact with obstacles or noxious stimuli, 32 out of 101 *lov^47^* larvae show bouts of spontaneous avoidance of this type in the absence of any obvious trigger (Figure 8 B). Feeding activity, as assessed by counting the rate of mouth hook movement associated with food shoveling, is also depressed (Figure 8 D). Given that reduced feeding could result from decreased chemosensory input from food, we investigated food-related sensory capacity in these larvae. Although *lov^47^* larvae are slow to respond in taste tests, due to their decreased mobility, their preference for sucrose versus sucrose laced with caffeine (a repellent stimulus) is indistinguishable from wild type (data not shown) and they show a normal preference for fructose (a strongly attractant stimulus) versus lactose (a non-stimulating sugar [27])(Figure S1). These data suggest that poor motor control rather than reduced food sensing could underlie the “failure to thrive” of *lov^47^* larvae.

The early embryonic Lov expression pattern is normal in *lov^47^*, but major defects in the late neural expression precede the behavioral problems seen in *lov^47^* larvae. Expression of *lov* transcript D during neural differentiation is highly suppressed (Figure 3) and Lov staining is missing from many neurons in both the CNS and PNS. In the abdominal PNS, staining with the panneural antibody 22C10 established that the relevant neurons were present but no longer expressing Lov (Figure 6 D). We also established that the mutation has differential effects on Lov expression amongst the various Lov staining neurons identified. For most of the eso neurons and tracheal neurons, v′td1 and v′td2, Lov expression is not dramatically altered. But strikingly, expression in lch2 and 4 is absent in most segments (Figure 6 E′, E′′). Thus, the *lov^47^* deletion appears to specifically affect regulatory elements that direct expression in these neurons.

**Figure 9:**
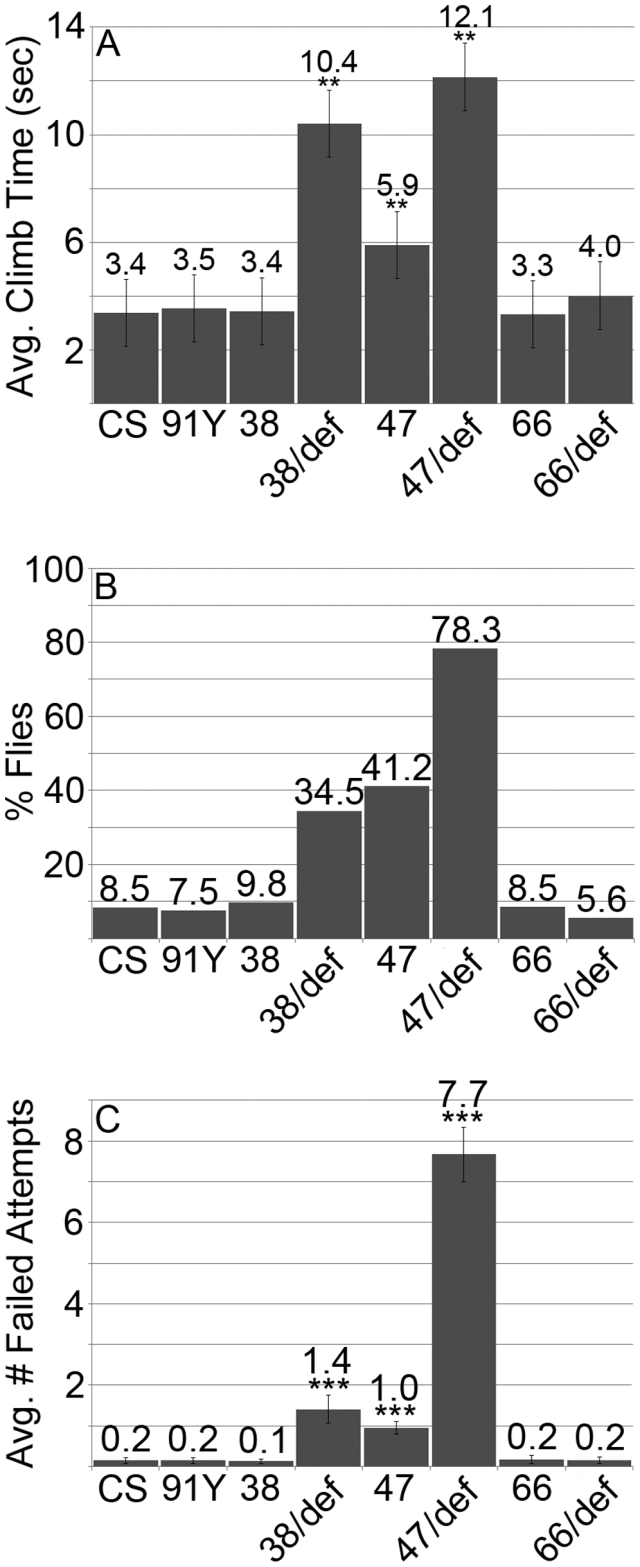
Negative gravitactic defects in *lov^38^* and *lov^47^* adults. **A** - Climb times in the negative gravitactic climb assay (see Material and Methods) for flies of each genotype that completed the climb in less than one minute. **B** - Percentage of flies of each genotype that failed to complete the climb in at least one trial. **C** - The average number of failed climbs for the 10 consecutive trials performed for each fly. CS = Canton-S, 91Y = *lov^91Y^*, 38 = *lov^38^*, 47 = *lov^47^*, 66 = *lov^66^*, def **-** *lov* deficiency chromosome *SB1*. Statistics as previously. ** = p<0.01, *** = p<0.001 as compared to CS.

*lov^47^* adults were tested for responses to light, attractive and repellent tastes, repellent odors and water. Their responses in these assays were essentially wild type (Figures S2–S5) suggesting, as for *lov^47^* larvae, that many sensory pathways are intact. However, two major behavioral defects were detected. When assayed in a negative gravitactic climb test, *lov^47^* homozygous and hemizygous adults showed two defects. For flies that completed climb assays, the climb rate was slower than for controls, with *lov^47^* hemizygotes taking three times as long to complete the 5 cm climb, as a result of pausing and non-upward movement (Figure 9 A). But in addition, *lov^47^* mutant flies showed a failure to complete climb tests, either from failure to initiate climbing or cessation of climbing during the assay. Each individual fly was sequentially tested 10 times and ∼40% of homozygotes, ∼80% of hemizygotes, failed to complete the climb in at least one of these repetitions (Figure 9 B). The average percentage of failed climbs per fly for hemizygotes was particularly high (76%, Figure 9 C). *lov^47^* hemizygous adults showed poor viability, typically dying within five days of eclosion.

**Figure 10:**
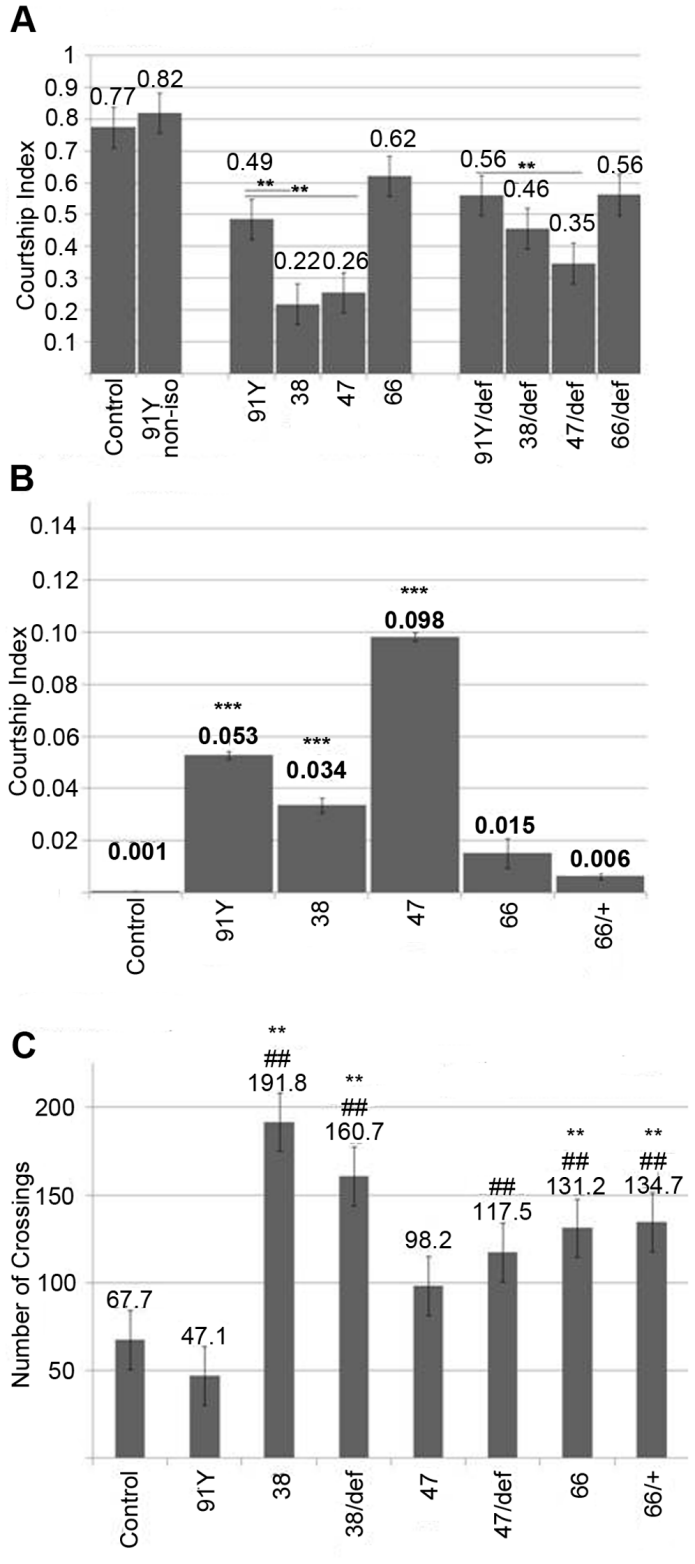
Courtship defects in *lov^38^* and *lov^47^* males. **A -** Directed courtship. Courtship indices for courtship directed towards the female. The courtship indices for *lov^91Y^* in its original genetic background (91Y non-iso) and after isogenization of *lov^91Y^* into the same *w^+^* background (see text) as the other *lov* mutants (91Y) are shown. Comparison of these two indices demonstrates that the new genetic background suppresses courtship significantly. Courtship behavior for *lov^38^*, *lov^47^* and *lov^66^* is therefore compared only to the *lov^91Y^* iso line (91Y) to correct for this background suppression of courtship. Statistics as previously. ** = p<0.01 as compared to isogenized *lov^91Y^*. **B -** Non-directed courtship. Courtship indices for elements of the courtship ritual performed while not pursuing the female. Statistics as previously. *** = p<0.001 as compared to Canton-S control. **C -** Locomotor activity for males alone in courtship chambers (see Material and Methods). Control - Canton-S, 91Y = *lov^91Y^*, 38 = *lov^38^*, 47 = *lov^47^*, 66 = *lov^66^*,+ = *CyO* chromosome, def - *lov* deficiency chromosome *SB1*. Statistics as previously. ** = p<0.01 as compared to Canton-S control. ^##^ = p<0.01 as compared to isogenized *lov^91Y^.*

*lov^47^* males also showed strong defects in male courtship. Normal courtship was significantly lower than for males of the parent *lov^91Y^* genotype, which showed wild type courtship in its original genetic background (see legend Figure 10 A). In addition, *lov^47^* males showed “non-directed” courtship, performing elements of the courtship ritual without focusing their behavior on the female. This was particularly true of wing extension. Males could be seen wandering around the courtship chamber rather than pursuing the female, performing wing extension as they did so. Separate Courtship Indices for “directed” (Figure 10 A) and “non-directed” courtship (Figure 10 B) were calculated for *lov^47^* and other genotypes. These revealed a low level of non-directed courtship for the parent *lov^91Y^* chromosome and for the related *lov^38^* deletion (Figure 10 B and see below). Further, this quantitation established that for *lov^47^*, almost 30% of **total** courtship activity is of the “non-directed” type (Figures 10 A, B). Due to their poor viability, only a limited number of *lov^47^* hemizygous males could be tested for courtship at seven days of age. These males tended to be immobile in the presence of females and non-directed courtship was largely replaced by “passive” courtship, where males would only execute elements of the courtship routine if the female came very close. However, a generalized defect in locomotion was not a factor in lowering the courtship index for these males. Activity assays performed in the courtship chambers in the absence of females revealed that both *lov^47^* hemizygotes and homozygotes were at least as active, if not more so, than controls (Figure 10 C).

#### *lov^38^* mutants show adult behavioral phenotypes similar to those of *lov^47^*

The deletion associated with *lov^38^* is contained within the *lov^47^* deletion, sharing the same downstream breakpoint as *lov^47^*. Unlike *lov^47^*, *lov^38^* does not affect the late embryonic expression pattern of Lov protein and *lov^38^* mutant larvae appeared and behaved like controls (Figure 8 C, D). However, *lov^38^* does show adult phenotypes similar to those of *lov^47^*. Male courtship is decreased to levels comparable to those seen for *lov^47^* males (Figure 10 A) and is accompanied by the same kind of non-directed courtship activities (Figure 10 B). *lov^38^* also fails to complement the *lov^47^* defects in male courtship (data not shown). *lov^38^* adults show poor climbing ability although the defects are not as marked as those of *lov^47^* (Figure 9). However, the general locomotor activity shown by *lov^38^* males when alone in the courtship chamber is significantly higher than that of *lov^47^* males (Figure 10 C).

#### *lov^66^* is a neomorphic mutation affecting oogenesis

In *lov^66^*, a different region of the locus is deleted than in *lov^38^* and *lov^47^* and the phenotypic consequences are markedly different: *lov^66^* produces female sterility (**Figure S6**). Eggs from homozygous or hemizygous *lov^66^* mothers have a low hatch rate (∼20%) irrespective of mating partner genotype (**Figure 7**) and the mutation has to be maintained as a balanced stock. Hoechst staining established that ∼80% of the unhatched eggs showed no development and were apparently unfertilized. This lack of development did not correlate with problems in mating or sperm storage since *lov^66^* homozygous females are courted by Canton-S males as vigorously as Canton-S females (data not shown) and after mating, *lov^66^* females carry stored sperm in their seminal receptacles as frequently as controls (**Table S1**).

**Figure 11:**
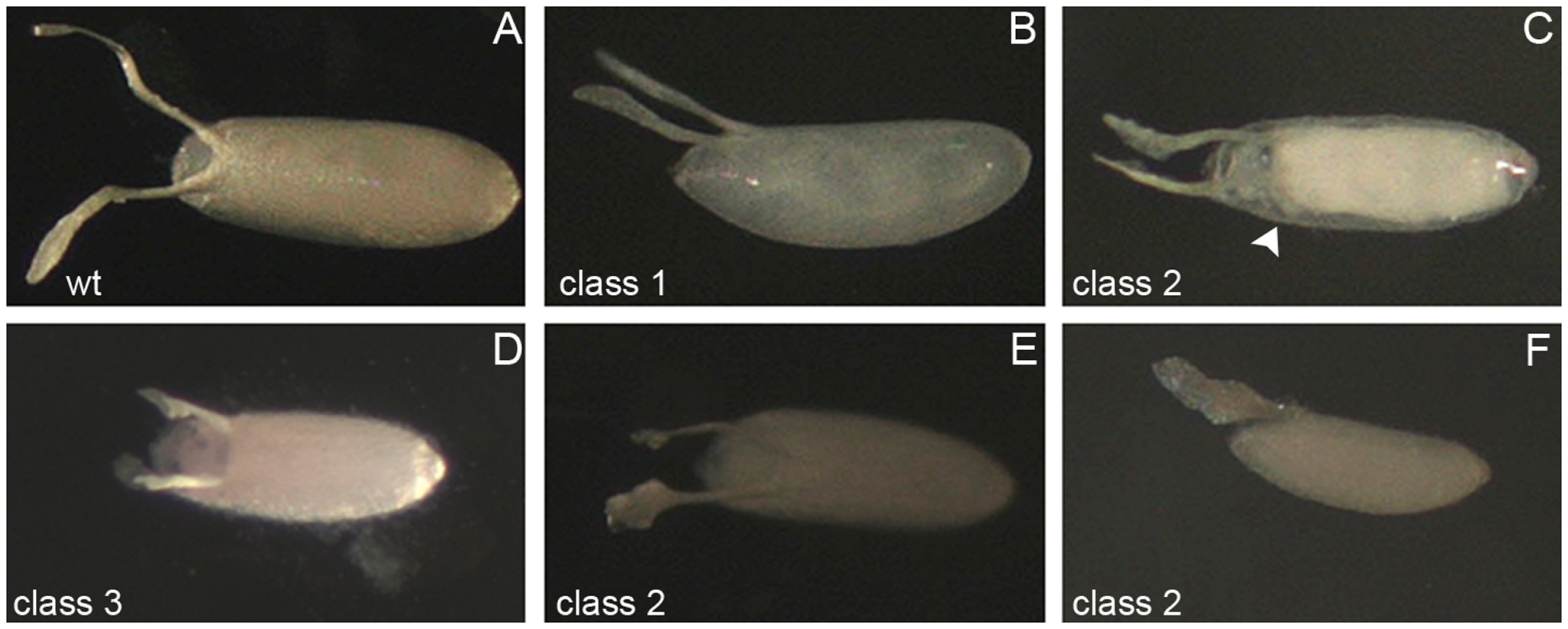
Classes of abnormal eggs produced by *lov^66^* mothers. **A** - wild type egg with opaque chorion and two opaque dorsal appendages (DAs). **B-F** - examples of abnormal eggs from *lov^66^* homozyous or hemizygous mothers. **B** - class 1 egg (see text) with translucent chorion and translucent, weak DAs. **C** - class 2 egg (see text). DAs are abnormal and translucent and formation of the anterior chorion has failed. Illuminated to show chorion-like barrier (arrow) that has prevented complete nurse cell dumping. **D** - class 3 egg. Chorion is opaque and thick but egg is short, with stubby DAs. **E, F** - class 2 short, flaccid eggs with translucent chorions and translucent abnormal DAs.

Eggs from *lov^66^* homozygous or hemizygous mothers show a range of defects. Some aberrant eggs (Class 1, Figure 11) were of normal shape and size, but had a transparent, thin eggshell (chorion) and dorsal appendages (DAs) and were typically somewhat flaccid. This phenotype has been well described [28] and illustrated previously (compare Figure 1 B of Schwed et al. [29] with Figure 11 B) and results from failure to amplify or express chorion protein genes late in oogenesis [30], [31]. However, in addition to a thin chorion, some eggs also showed defects in the final stages of egg morphogenesis (Class 2, Figure 11). Aberrantly shaped DAs were seen, often on eggs showing failed formation of the anterior-most dorsal structures of the eggshell. These eggs often appeared unsealed at the anterior and absorbed color from the grape plates used for egg collections. Late in oogenesis the germline nurse cells “dump” their cytoplasmic contents into the oocyte proper. For *lov^66^*, eggs in which the chorion appeared to seal off the nurse cell compartment prematurely and prevent complete dumping were identified (Figure 11 C). A further abnormal egg class (Class 3, Figure 11) consisted of short eggs with sealed eggshells showing a characteristic cup-shaped indentation at the position of the operculum flanked by two stubby branched DAs. In most cases the chorion and DAs on these eggs were thick and opaque; a few short eggs of this type also showed the transparent chorion/DA phenomenon and were categorized as Class 2 eggs. Finally a small number of eggs appeared otherwise normal except for aberrantly shaped DAs (Class 4).

An analysis of oogenesis established that defects in egg chamber formation for *lov^66^* mothers were limited to the late stages of oogenesis. Follicles prior to the vitellogenic stages appeared morphologically normal and markers for specialized somatic cells associated with the follicles, such as the stalk and polar cells, showed that these cells were present in normal numbers and at their normal positions (data not shown). However, abnormalities consistent with the defects seen in laid eggs were detected during the vitellogenic and later stages, including malformed DAs and incomplete nurse cell dumping associated with premature formation of a chorion-like structure between the nurse cells and oocyte proper. Overall, these findings show that *lov^66^* affects late events in egg morphogenesis including aberrant activity of the somatic cells that form the chorion and associated structures.

**Figure 12:**
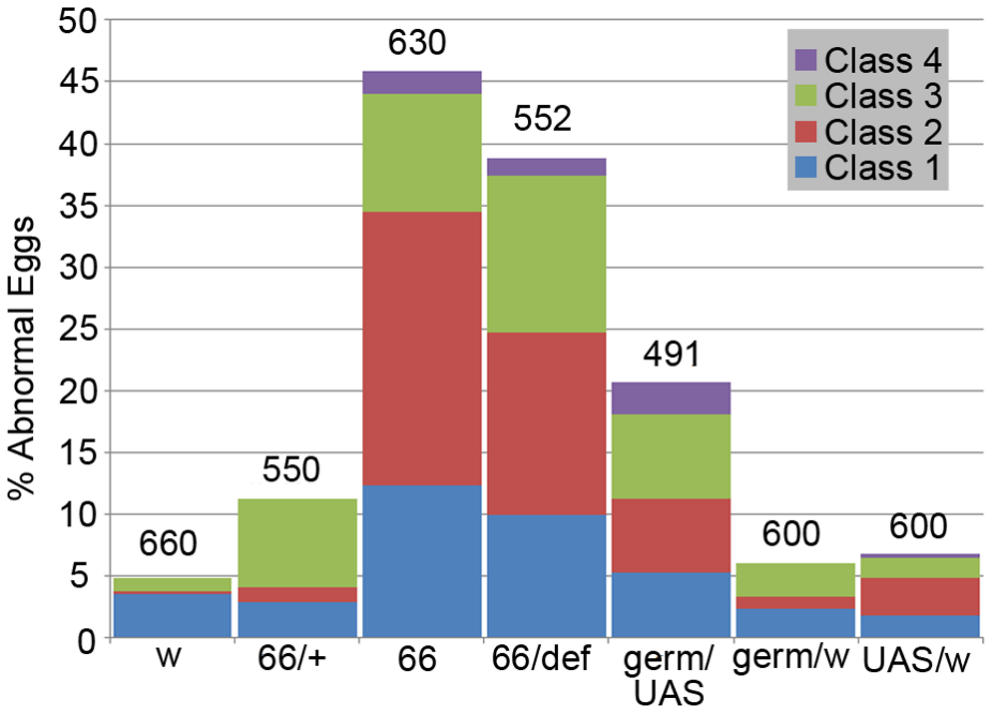
Abnormal egg production in *lov^66^*-related genotypes. The percentage of abnormal eggs of various classes (see text) is shown for *lov^66^* homozygous (66), heterozygous (66/+ = 66/CyO), and hemizygous mothers (66/def), for mothers (germ/UAS) expressing UAS-*lov* ectopically in the ovarian germ-line under the GAL4 driver P (GAL4::VP16-nos.UTR) CG6325^MVD1^ and for appropriate control mothers (w = *w^1118^*, germ/w and UAS/w). All females were mated to *w^1118^* males. Numbers of eggs examined for each genotype are shown above the bars.

These defects were surprising given that we could not detect either *lov* transcripts (Figure 2) or Lov protein in wild type egg chambers. RT-PCR analysis of *lov* transcript expression in the *lov^66^* mutation resolved this discrepancy. As shown in Figure 2, in contrast to the control situation, all four *lov* mRNAs are detectable in the *lov^66^* ovary. Thus the *lov^66^* ovarian phenotype involves ectopic expression of *lov* in this tissue. To determine if deliberate ectopic expression of *lov* in the germline or somatic follicle cells of the egg chambers could produce effects comparable to *lov^66^*, we used the GAL4-UAS system [16] to drive *lov* expression in both cell types. We found that expression of *lov* in the female germline using the GAL4 driver P(GAL4::V P16-nos.UTR) CG6325^MVD1^ [32] reproduced the *lov^66^* phenotype. Although a lower fraction of the eggs were abnormal (Figure 12), qualitatively the same range of aberrant egg phenotypes was produced by GAL4::V P16-nos.UTR) CG6325^MVD1^/UAS-*lov* mothers. The quantitative difference could reflect a lower level of ectopic *lov* expression than that seen with *lov^66^* itself. We tested three GAL4 follicle cell drivers, c532 [33], c204 and c355 [34] in an attempt to determine whether ectopic *lov* expression in the somatic cells also affects egg development but, presumably as a result of ectopic expression of *lov* in other tissues, these crosses produced essentially no adult survivors making meaningful analysis impossible.

We deduce that these *lov^66^*-associated maternal effects on oogenesis result from the ectopic expression of *lov* in the ovary. However, surprisingly, whereas *lov^66^* hemizygous mothers show comparable maternal effects on hatch rate and eggshell defects (Figures 7, 12), eggs from *lov^66^* heterozygous mothers (*lov^66^*/*CyO*) have largely normal egg cases and show normal hatch rates. This finding, which may indicate a transvection effect at the locus (see Discussion), allowed us to assess the effects of the *lov^66^* mutation on embryonic viability in the absence of the *lov^66^* maternal effect. As shown in Figure 7, *lov^66^*/*CyO* × *lov^66^*/*CyO* crosses have an essentially wild type hatch rate, when corrected for the death of *CyO/CyO* progeny, indicating that *lov^66^*/*lov^66^* embryos are viable. To determine whether, like *lov^47^*, *lov^66^* affects the zygotic Lov protein expression pattern we examined *lov^66^* embryos from a *lov^66^*/*CyO-GFP* stock. Given that GFP expression from the balancer chromosome develops slowly, we could only reliably examine the later neural expression of Lov. In contrast to *lov^47^* embryos, *lov^66^* embryos showed no loss in Lov expression in the PNS or major changes in CNS expression (data not shown). The resultant larvae grew as well as controls when selected from the balanced *CyO-GFP* stock and grown as homozygotes in yeast paste. These larvae also showed none of the larval behavioral defects associated with the *lov^47^* mutation (Figure 8). When *lov^66^*/*CyO* stocks were grown in uncrowded conditions, *lov^66^* homozygous adults emerged in the expected ratio (that is 2 *lov^66^*/*CyO* : 1 *lov^66^*/*lov^66^*) but the number of emergent *lov^66^* homozygotes dropped considerably in crowded conditions, suggesting some growth disadvantage in the presence of heterozygous siblings. The lower expression of *lov* transcript B during larval and early pupal life (Figure 3) may contribute to this effect.

*lov^66^* homozygous adults were subjected to the same array of behavioral tests as *lov^47^* and *lov^38^* and showed none of the behavioral problems seen for those mutants. Climbing behavior and male courtship were comparable to controls (Figures 9 and 10) and *lov^66^* males were essentially normal in the sensory assays we performed (Figures S2–S5), with the exception of a statistically significantly slight reduction in response to sucrose as a tastant (Figure S3).

## Discussion

### The Lov Early Embryonic Expression Pattern

The early Lov staining pattern suggests roles for the protein in three aspects of early embryogenesis. The intense staining in rows of cells that form the boundaries of transient folds in the ectoderm may indicate that Lov functions to promote the mechanical properties of these cells, supporting their role in global re-structuring of the embryo. The strong staining in the dorsal head region, which spans the procephalic neural ectoderm, suggests a role in determination of neural lineages within the head. Finally, the early expression in the amnioserosa could signify a role for Lov in the early differentiation of this extraembryonic tissue and thus its function in dorsal closure and germ band retraction [35], [36]. None of the mutations characterized here is a null and none of them affects these early expression elements. Experiments with *lov* RNAi are therefore being used to address the functions of these early patterns.

By comparing the Lov expression pattern to that of other early acting genes, we have identified loci that may be upstream regulators of *lov*, or act in parallel with it, during these early stages. The three highly-related Dorsocross proteins, which all have the same expression pattern, show a strikingly similar distribution to Lov at stage 8, with the same strong head and amnioserosal staining [37]. This set of proteins does not show the stripe elements seen for Lov at cellular blastoderm but the mRNA expression patterns for *pannier* (*pnr*) [38], which encodes a GATA transcription factor [39] and for a gene encoding the serotonin receptor 5HT2 [40] both consist of broad saddles of stripes across the dorsal midline that overlap the Lov saddle along the anterior/posterior axis. Interestingly the 5HT2 receptor has already been shown to play a role in transverse furrow formation in the ectoderm during germ band extension [41]. *pnr* and the *Dorsocross* genes are both required for formation of the amniserosa and are regulated by *decapentaplegic* (*dpp*) and *zernknullt* (*zen*), which act early in the formation of the dorsal embryonic tissues. Both the Zen protein [42] the *dpp* mRNA pattern [43] also show intense staining in cells at the boundaries of the transient ectodermal folds, as seen with Lov, suggesting that these early acting regulators also control the Lov pattern. The stripe component of the early Lov pattern evokes comparisons to the pair rule and segment polarity genes, whose roles in early pattern formation entail expression in distinct stripes along the anterior-posterior axis [44]. The striped expression of the serotonin receptor 5HT2 is already known to be regulated by the pair rule gene *fushi tarazi* (*ftz*) and its ubiquitously expressed cofactor, *ftz-F1* [45]. We plan epistatic analyses to determine the position of *lov* in these early genetic hierarchies.

### The Lov Abdominal PNS Expression Pattern

The timing and expression pattern of Lov in the PNS indicates that *lov* acts late in neural development to direct final neuronal differentiation of subsets of neurons. Given the expression pattern of Lov within the various classes of sensory neurons, we can predict some elements of the transcriptional hierarchies that regulate *lov* in the PNS. Thus the *lov* expression in most eso neurons indicates that *lov* is activated downstream of *achaete* and *scute*, the proneural genes of the *achaete-scute complex* (ASC) that direct formation of the eso mother cells [46] and downstream of *cut*, which maintains the “eso” identity in the mother cell lineage [47]. The da class of multiple dendritic neurons, which does not express Lov, also derives from these lineages [48] and so additional regulators must act to prevent *lov* expression in these neurons. Similarly the absence of Lov in the bp multiple dendritic class suggests suppression of *lov* by *amos*, the proneural regulator of this class [49].

The expression pattern of Lov within the chordotonal lineages indicates considerable complexity in *lov* regulation. Limitation of Lov expression to two neurons of the ch class (neurons 2 and 4 of the lateral chordotonal five-neuron cluster) is a striking finding. To our knowledge this is a unique observation that suggests for the first time functional differences amongst the five chordotonal organs (CHOs) at this site. Developmentally, these CHOs are already known to originate via two distinct pathways [50]. Initially, three chordotonal precursors are formed under the direction of the proneural gene *atonal* [50], [51]. Subsequently *rhomboid* expression in these precursors leads to EGF receptor signaling, induction of *argos* in adjacent cells, and formation of two further chordotonal precursors. It is not known precisely which of the final five lateral CHOs correspond to the two derived from the second phase of development but one possibility is that they are the two Lov expressing neurons. Lov expression would thus be downstream of both *atonal* and EGF signaling in these neurons.

One of the two CHOs of the ventral cluster (VchA) is also induced by the later wave of EGF signaling activity [50], but we find that neither of the two ventral CHOs (VchA and B) expresses Lov, indicating different regulatory mechanisms when compared to lch2 and 4. Further, both the VchA and VchB neurons undergo an additional cell division to generate the two md tracheal neurons td1 and td 2 [48]. These neurons, in contrast to their sibling VchA and VchB neurons, express Lov strongly. Generation of these varying Lov expression patterns within the CHO lineages clearly requires a multiplicity of regulatory mechanisms.

The analyses of *lov* mRNA expression in the midline lineages performed previously [3]–[6] indicate that regulation of *lov* within the CNS is also dynamic and complex and that here too *lov* has roles in the final differentiation of particular neuronal sub-types. The single cell per segment that first expresses Lov protein in the CNS (Figure 5) has been identified by the Crews lab as one of the eight primordial midline precursor cells whose determination is controlled by *singleminded*. Through stages 11–17 of embryogenesis *lov* mRNA is transiently expressed in the posterior midline glia, the median neuroblast (MNB) cell and a subset of ventral unpaired median motorneurons (VUMs) to give a final expression pattern at stage 17 in just three neurons: a single cell of the MNB progeny and the sister interneuron and motor neuron derived from division of midline precursor neuron 6 (MP6). *Notch* and *lethal-of-scute* act at various points to regulate these expression patterns [5], [6].

### The *lov^47^* and *lov^66^* Phenotypes Reflect Loss of Different Transcription Regulatory Elements

The *lov^47^* and *lov^66^* mutations produce strikingly different phenotypes, with *lov^47^* mutants showing multiple behavioral defects and *lov^66^* mutants proving behaviorally unexceptional but strongly female sterile. These differences indicate that the mutually exclusive, non-coding, DNA sequences deleted in the two mutations have differing regulatory roles for the individual *lov* transcripts. Some of the effects on individual transcripts produced by each mutation generate the differing phenotypes detected here but other effects are without consequence, at least in terms of the traits we assayed. Thus the DNA deleted in *lov^47^* has a strong positive role in production of *lov* transcript D, a lesser positive role in production of transcript A and a negative role in production of transcript C (Figures 2 and 3), but the phenotypic effects detected here all appear to have their origins in the loss of transcript D alone. Interestingly, transcript B, which has the same transcription start site and expression pattern as transcript D, is not affected by the deletion. It is possible therefore that the embryonic neurons retaining Lov expression in the *lov^47^* mutant are neurons that express transcript B as opposed to transcript D.

We hypothesize that a global repressor of *lov* transcript expression in the female gonad is deleted in the *lov^66^* mutation and the main phenotypic consequences of this mutation result from loss of this DNA. The deleted DNA also contributes in a positive way to expression of transcripts A, B and D in various tissues and stages (Figures 2 and 3). Due to the female sterility, we did not examine transcript D levels in late embryogenesis for *lov^66^* by RT-PCR, but Lov protein expression appeared normal in these stages. However, in adults, *lov^66^* depresses neural transcript D expression even more strongly than *lov^47^*. Given their marked differences in terms of behavioral consequences, we conclude that the two mutations affect transcript D expression in different subsets of adult neurons. As discussed below, a component of the *lov^66^* induced female sterility may result from loss of *lov* expression in neurons controlling the female genitalia that are unaffected by *lov^47^*.

### The *lov^47^* Behavioral Phenotypes

The larval phenotypes identified for *lov^47^* are defects in motor functions: the rates of food shoveling responses and forward locomotion are significantly decreased. The Crews lab has shown that loss of the ventral nerve cord midline neurons, which includes *lov* expressing cells, results in sluggish larval forward motion [5], suggesting that this *lov^47^* phenotype originates in the CNS. But *lov^47^* larvae also show bouts of backward locomotion, which are normally a stimulus-elicited avoidance response. A Central Pattern Generator (CPG) within the CNS coordinates the rhythmic locomotor contraction waves of the larval body wall and prior work has shown that loss of sensory input from the periphery disrupts CPG performance, producing both decreased forward, and spontaneous backward, contraction waves [52]. Caldwell and Eberl have further provided evidence that most of this peripheral sensory feedback to the CPG derives from the chordotonal neurons of the PNS [53]. Thus, the specific loss of Lov expression in two of the five lateral chordotonal organ neurons in *lov^47^* could diminish sensory feedback to the CPG and contribute to abnormal backward movement.

Motor defects are also seen in *lov^47^* adults. The defective *lov^47^* gravitactic climb responses cannot be considered a consequence of general sluggishness since *lov^47^* adults show enhanced locomotor activity when alone in courtship chambers. They appear to represent a more severe form of the diminished negative gravitaxis produced by the original *lov^91Y^* mutation. Thus the original *lov^91Y^* mutant has normal climb behavior (Figure 9 and [7]) and its decreased negative responses to gravity are only uncovered in the gravitactic maze assay [7]. Like the *lov^47^* larval locomotor defects, the *lov^47^* defective gravity responses could also reflect loss of *lov* function in chordotonal neurons. Although the enhancer trap *P{GawB}* insertion of *lov^91Y^* does not show GAL4 expression in Johnston’s organ, a major graviperceptor organ in the antenna, GAL4 is expressed in the adult leg femoral chordotonal organ (data not shown), another proprioceptor organ with a demonstrated role in gravity responses in other insects (reviewed in [54]). Thus, the loss of negative gravitaxis in adult *lov^47^* mutants could reflect deletion of enhancer sequences adjacent to the *lov^91Y^ P{GawB}* insertion point that promote *lov* expression in the femoral chordotonal organ.

The larval defects of *lov^47^* indicate loss of central organization of locomotor responses. The aberrant courtship responses of *lov^47^* males reinforce this concept of failed coordination of innate responses. Male courtship is highly stereotypic with a series of ordered steps (following the female, tapping her, wing vibration, licking the female) leading to abdomen curling and copulation. *lov^47^* males showed a marked inability to pursue females continuously but still performed non-directed wing extension, and occasionally abdomen curling, often towards the courtship chamber walls. As noted earlier, Lov belongs to a class of putative transcription factors that includes Fruitless (Fru), the master regulator of male courtship behavior. Given that this class of transcriptional regulators is known to form homo- and hetero-dimers, it is tempting to speculate that Lov might affect courtship responses through protein-protein interaction with Fru. We have not yet attempted a detailed analysis of Lov protein expression in the adult head but preliminary experiments indicate that Lov is expressed in a very large number of adult CNS neurons, a subset of which also express Fru (data not shown).

### The *lov^66^* Neomorphic Phenotype

The *lov^66^* induced female sterility appears to represent a neomorphic phenotype resulting, at least in part, from ectopic expression of *lov* in the ovarian germline. However, some aspects of our data for this phenotype are puzzling. First, although *lov^66^* hemizygotes show the same phenotype as homozygotes, *lov^66^* heterozygotes are essentially wild type both with respect to eggshell features and egg hatch rate (Figures 7 and 12). Given that the single copy of the mutant chromosome present in both hemizygotes and heterozygotes should be capable of deregulated *lov* expression, this result is unexpected. The two genotypes differ however in the state of the *lov* locus on the homolog paired with the *lov^66^* chromosome. In hemizygotes, the entire locus is missing whereas in heterozygotes the presumed regulatory DNA is present on the homolog and could regulate expression from the *lov^66^* chromosome via transvection. Recently transvection has been shown to be a common regulatory mechanism in Drosophila [55], supporting this possibility.

A further puzzling comparison involves the hatch rates and eggshell defects associated with *lov^66^* homozygous/hemizygous mothers and mothers expressing UAS-*lov* under the ovarian germline GAL4 driver. Whereas for mothers expressing UAS-*lov* in the germline, the fraction of aberrant eggs (20%) correlates well with the fraction of unhatched eggs (20%), for *lov^66^* mothers, the fraction of unhatched eggs (∼80%) is significantly higher than the fraction of detectably abnormal eggs (∼45%). Given that the unhatched eggs from *lov^66^* mutant mothers appear undeveloped and unfertilized, one possible explanation is that *lov^66^* can disrupt egg maturation and fertilization through a second route, involving other elements of the female reproductive system. Adult females lacking the midline CNS neuron population (which includes *lov* expressing neurons) show sterility thought to reflect loss of innervation of the female genitalia, perhaps producing failed fertilization [5]. It is possible that *lov^66^* causes loss of elements of *lov* CNS expression in addition to producing ectopic ovarian expression.

Mechanistically, ectopic expression of *lov* in the ovary must be presumed to produce interference with the action of one or more factors active in eggshell synthesis and morphogenesis. Making the simplest assumption that a single factor is affected by Lov to produce both phenotypes, we sought to identify regulators that affect both eggshell synthesis and eggshell morphogenesis and for which action in the follicle cells is controlled by signals from the germline. Two transcriptional regulators, Broad [33], [56] and Tramtrack [57]–[59], meet these criteria. Interestingly these are both BTB transcriptional regulators, offering the possibility, as discussed for Fruitless above, that Lov misregulates function by direct protein interaction. For both Broad and Tramtrack, there is evidence that expression and/or action in the somatic follicle cells is controlled by ecdysone signaling from the germline [58]–[60]. Further, mutation of *broad* and dominant interference with ecdysone signaling produce eggshell phenotypes similar to those seen with *lov^66^*. In particular i) the fragile chorions and short/malformed/branched DAs from mothers mutant for the *rbp* class of *broad* mutations [56] are similar to the eggs from *lov^66^* mothers and ii) the short eggs with stubby DAs and cupshaped dorsal anteriors produced by dominant negative inhibition of ecdysone receptor function in the follicle cells, or loss of ecdysone production, are like the Class 3 eggs identified for *lov^66^* [61]. These findings suggest that the neomorphic action of Lov in the ovarian germline affects ecdysone signaling and that downstream effects on follicle cell function include disruption of the action of Broad. Although we have not pursued the underlying mechanism of this neomorphic *lov^66^* phenotype in detail, we have determined that, in ovaries from *lov^66^* mothers, effects on *broad* are not limited to effects at the level of protein/protein interaction. *broad* transcription is depressed in *lov^66^* ovaries in addition to disruption of Broad protein localization (Figure S7). Depression of *broad* expression is consistent with the depressed *broad* expression seen on loss of ecdysone signaling.

### Note Added in Proof

In addition to lacking transcript A (RA), the Flybase page for CG16778 now shows a new minor *lov* transcript (transcript RE), which was not studied here and which contains additional 3′ protein coding sequences.

## Supporting Information

Figure S1 ***lov^47^*** **larvae show normal taste responses to sugars.** Petri dishes filled with two adjacent semicircles of 1% agarose gel, one with 10% fructose (strongly attractant) and red food coloring and the other with 10% lactose (non-stimulating) and blue food coloring, were used to test larval sugar preference. Twenty-five larvae, collected 65 hours after egg laying, were placed on the boundary between the two sugars and the number of larvae at the boundary and on each of the two sugar semicircles was counted after 5, 10 and 15 minutes. The experiment was repeated eight times (200 larvae of each genotype total) and mean values with standard error for all eight repeats are shown. A. Control Canton-S larvae. B. *lov^47^* larvae. Although *lov^47^* larvae take longer to move to the compartment of their choice, their preference for fructose at 15 minutes is indistinguishable from that of the controls. Error bars represent standard error. Assay modified from Xu et al., Nature Neuroscience 11, 676–682, 2008 and Schipanski et al., Chem. Senses 33, 563–573, 2008.
(DOCX)

Figure S2 ***lov*** **mutants show normal responses in a fast phototaxis assay.** The various *lov* mutants were tested for their ability to detect and respond to light. Flies were placed in a t-test apparatus in which one tube was exposed to a light source and one tube remained in the dark. The flies were allowed one minute to choose which tube to enter and then the number of flies in each tube was counted. For this assay, vials of up to 25 newly eclosed males were collected and allowed to age for three to five days before testing. The assay was repeated up to six times. Wild type flies show positive phototaxis. The responses of all *lov* mutants are indistinguishable from the control line (df 5; χ^2^ = 5.17; Control  =  Canton S n = 100, l*ov^91Y^* n = 170, *lov^38^* n = 132, *lov^47^* n = 82, *lov^66^* n = 138, *lov^66^/CyO* n = 146).
(DOCX)

Figure S3 ***lov^66^*** **has a decreased response to sucrose.** The proboscis extension response (PER) assay of Gordesky-Gold (Chemical Senses 33, 301–309, 2008) was used to investigate adult taste responses. Newly eclosed males were starved overnight in food vials with paper tissue moistened with water. Flies were then mounted on toothpicks with Tissue Tack and allowed to recover for three hours. Prior to testing, flies were satiated with water to ensure that tastant, and not water, responses, were assayed. Attractive (1% or 4% sucrose) or repellent (4% caffeine +4% sucrose) tastants were touched to the front legs and the proboscis response noted. Flies were tested three times, and given water between testings. A score of one (“tasting”) was given if a fly extended its proboscis all three times. Two additional trials were performed for flies with mixed responses in the first three trials. After five trials, any fly that extended its proboscis three or more times was scored as “tasting”. Approximately 50 flies from each line were tested. A chi square test was used to determine significance relative to the control, Ore R flies. The reduced response of *lov^66^* to sucrose and stronger response of *lov^91Y^* to sucrose+caffeine are statistically significant. (4% Sucrose: df 5; Ore R n = 50, *lov^91Y^* n = 50, *lov^38^* n = 50, *lov^47^* n = 50, *lov^66^* n = 47 (χ^2^ = 56.7), *lov^66^/CyO* n = 44 (χ^2^ = 56.7); 1% Sucrose: df 5; Ore R n = 50, *lov^91Y^* n = 50, *lov^38^* n = 50, *lov^47^* n = 48, *lov^66^* n = 47 (χ^2^ = 50.2), *lov^66^/CyO* n = 44 (χ^2^ = 50.2); 4% Sucrose and 4% Caffeine: df 5; Ore R n = 50, *lov^91Y^* n = 50 (χ^2^ = 13.5), *lov^38^* n = 50, *lov^47^* n = 50, *lov^66^* n = 51, *lov^66^/CyO* n = 51).
(DOCX)

Figure S4 **Olfactory responses to a repellent odorant for** ***lov*** **mutants.** Newly eclosed males were collected, aged for four to six days, then placed in empty food vials in groups of five. A Q-tip pre-soaked with 100 µl of repellent odorant (1% or 0.1% benzaldehyde) was then placed in the vial so that the tip was in the middle of the vial. For one minute, the number of flies at the far end of the vial (opposite the plug) was counted every five seconds. The number of flies at the far end was averaged to give an Olfactory Response Index (ORI). The assay was repeated ten times for each genotype. An ORI above 3 (above the red line) means flies are repelled by the odorant. A one-way ANOVA was performed to determine if there were differences among the lines. A Dunnett’s Test was used to compare the results of the mutant lines to the control, Ore R. Error bars represent standard error. Responses of the *lov* mutants to 1% benzaldehyde are indistinguishable from the control, Ore R, response (1% benzaldehyde: p = 0.593, n = 50 for all genotypes). The slightly stronger responses of *lov^91Y^*, *lov^38^*, *lov^66^* and *lov^66^*/*CyO* to 0.1% benzaldehyde as compared to the control are statistically significant. (0.1% benzaldehyde: p = 0.004; n = 50 for all genotypes). Assay modified from Anholt and Mackay, Behav. Genet. 31,17–27, 2001.
(DOCX)

Figure S5 **Olfactory responses to a neutral odorant for** ***lov*** **mutants.** Newly eclosed males were collected, aged four to six days and then placed in an empty food vials in groups of five. A Q-tip pre-soaked with 100 µl of neutral odorant (water) was then placed in the vial so that the tip was in the middle of the vial. For one minute, the number of flies at the far end of the vial (opposite the plug) was counted every five seconds. The number of flies at the far end was averaged to give an Olfactory Response Index (ORI). The assay was repeated at least ten times so that a minimum of 50 flies were tested. An ORI between 2 and 3 (between the green and red lines) means flies are neutral to the odorant. A one-way ANOVA was performed to determine if there were differences among the lines. A Dunnett’s Test was used to compare the results of the mutant lines to the control, Canton S. Error bars represent standard error. The slight attraction to water shown by the control and the slight repulsion to water shown by *lov^47^* are statistically significant. (p≤0.001; Control = Canton S n = 125, *lov^91Y^* n = 50, *lov^38^* n = 50, *lov^47^* n = 100, *lov^66^* n = 50, *lov^66^/CyO* n = 50). Assay modified from Anholt and Mackay, Behav. Genet. 31,17–27, 2001.
(DOCX)

Figure S6 ***lov^66^*** **decreased fertility is attributed to the females.** Average total progeny number for the crosses shown above was determined as follows. Single male/single virgin female mating pairs were set up in vials and transferred on day 8 and then day 16 to new vials. On day 24 they were removed from the third vial and discarded. Adult offspring were then collected from each vial for eight days after eclosion of the first adult. A minimum of 10 mating pairs were scored for each line. Female *lov^66^* mutants have significantly decreased fertility when compared to *lov^66^* males in terms of producing viable progeny when mated to control flies. A one-way ANOVA was performed to determined to assess significance of differences between crosses. A Dunnett’s Test was performed with *w^1118^* as the control. Error bars represent standard error. *** = p<0.001 relative to the *w^1118^* control; M = males; F = females.
(DOCX)

Figure S7 ***lov^66^*** **affects Broad expression in the ovary.** In stage 10 of oogenesis, two patches of somatic follicle cells on the dorsal surface of the oocyte compartment show elevated nuclear expression of Broad (Tzolovsky et al., Genetics 153,1371–1383, 1999). This enhanced Broad expression commits the affected cells to dorsal appendage formation. A. The Broad patches (arrowheads) on a stage 10 wild type Canton-S egg chamber. Broad staining of the nurse cell nuclei (n) and oocyte nucleus (o) is artifactual and was intermittently seen with the antibody used. B. A normal size stage 10 egg chamber from a *lov^66^* mother. Although the overall pattern of Broad expression is similar to wild type, enhanced Broad expression at the position of the two presumptive patches of dorsal appendage cells is barely detectable. C. A short stage 10 egg chamber from a *lov^66^* mother. Again, overall patterning of Broad expression is relatively normal but expression within the patches is highly aberrant. D. Semi-Q RT-PCR to probe for all *broad* transcripts within ovaries from one-day-old control (*w^1118^*) and *lov^66^* homozygous females shows that Broad expression is depressed in *lov^66^* ovaries. **Methods. A-C.** Ovaries from Canton-S or *lov^66^* homozygous mothers were fixed in 4% paraformaldehyde and stained with the Broad-core monoclonal antibody 25E9-D7 (1∶250 dilution) from the Developmental Studies Hybridoma Bank and an Alexafluor-488 labelled goat anti-mouse secondary antibody (Invitrogen,1∶500 dilution). The Broad-core antibody recognizes sequences common to all isoforms of Broad. **D.** Semi-Q RT-PCR on ovarian RNA was performed as described in Material and Methods of the main text. Primers termed Broad F1 (5′ TGCAGGATGTCAACTTCATGGACC 3′) and Broad R (5′TATCTGAGCCAGATGGCTGTGTGT 3′), which span an exon-exon junction within the shared protein coding sequences of all *broad* transcripts, were used to probe for all processed *broad* transcripts. Actin 57B primers (see main text Material and Methods) were used in parallel to provide an internal control.
(DOCX)

Table S1 **Sperm transfer and storage is not impaired in** ***lov^66^*** **mutants.** A third chromosome *don juan*-*GFP* construct (Santel et al. Mech. Dev. 64, 19–30, 1997), which generates GFP-expressing sperm, was used to monitor sperm storage in the seminal vesicles and spermathecae of mated females. Males and virgin females were aged for 3–6 days after eclosion and set up in matings of the genotypes indicated. The sperm storage organs of females were dissected out after confirmed mating and were examined for GFP fluorescing sperm. There is no significant difference in the ability of *lov^66^* males to transfer sperm or *lov^66^* females to store sperm, relative to the *w^1118^* and *lov^66^*/*CyO* controls. χ^2^ = 1.04.
(DOCX)
