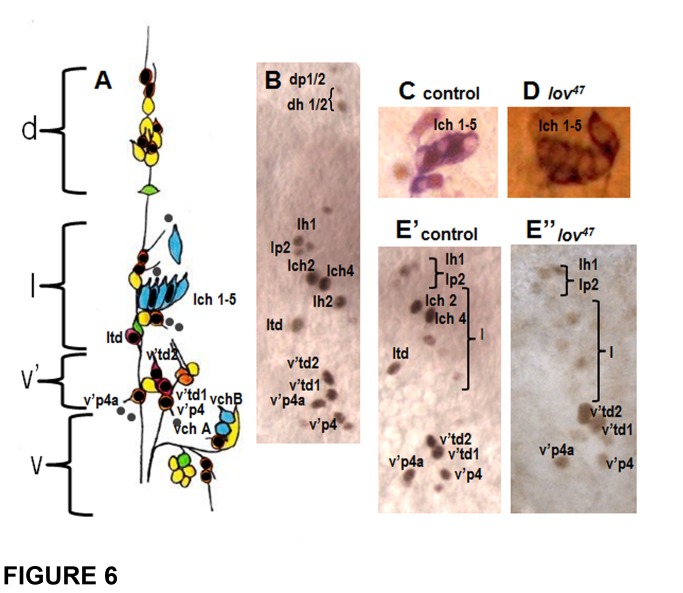# Correction: The Drosophila BTB Domain Protein Jim Lovell Has Roles in Multiple Larval and Adult Behaviors

**DOI:** 10.1371/annotation/88d89372-9fdc-48d7-83b3-26b61142a5e2

**Published:** 2013-08-06

**Authors:** Sonia M. Bjorum, Rebecca A. Simonette, Raul Alanis, Jennifer E. Wang, Benjamin M. Lewis, Michael H. Trejo, Keith A. Hanson, Kathleen M. Beckingham

The version of Figure 6 in the article is incorrect.

The correct version is available here: 

**Figure:**