# Supplementary figures and images for: The Drosophila BTB Domain Protein Jim Lovell Has Roles in Multiple Larval and Adult Behaviors
Source: PLoS One. 2013 Apr 19;8(4):e61270. doi: 10.1371/journal.pone.0061270 (PMC3631165; doi:10.1371/journal.pone.0061270)

A. Canton S


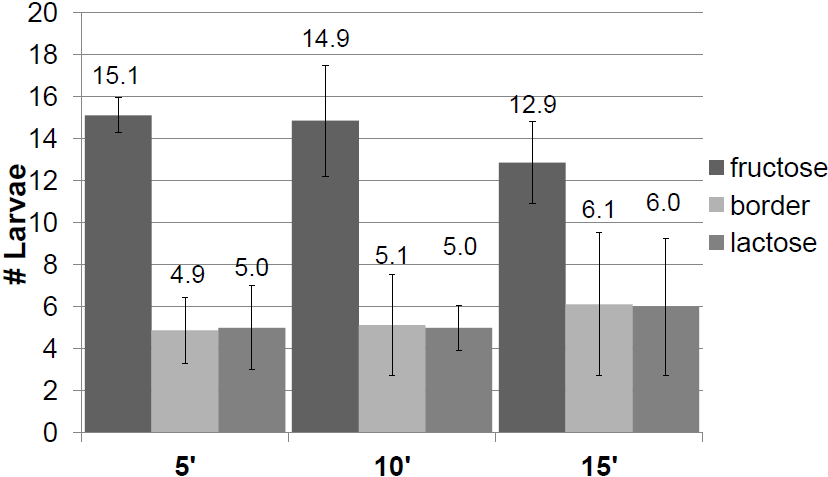


B. *lov47*


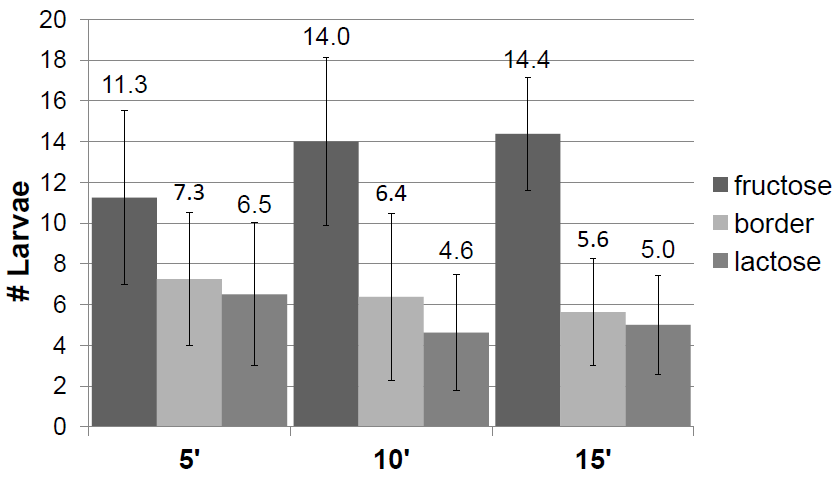


**FIGURE S1.**

Supplement: Figure S1 — lov47 larvae show normal taste responses to sugars. Petri dishes filled with two adjacent semicircles of 1% agarose gel, one with 10% fructose (strongly attractant) and red food coloring and the other with 10% lactose (non-stimulating) and blue food coloring, were used to test larval sugar preference. Twenty-five larvae, collected 65 hours after egg laying, were placed on the boundary between the two sugars and the number of larvae at the boundary and on each of the two sugar semicircles was counted after 5, 10 and 15 minutes. The experiment was repeated eight times (200 larvae of each genotype total) and mean values with standard error for all eight repeats are shown. A. Control Canton-S larvae. B. lov47 larvae. Although lov47 larvae take longer to move to the compartment of their choice, their preference for fructose at 15 minutes is indistinguishable from that of the controls. Error bars represent standard error. Assay modified from Xu et al., Nature Neuroscience 11, 676–682, 2008 and Schipanski et al., Chem. Senses 33, 563–573, 2008. (DOCX) [file pone.0061270.s001.docx]

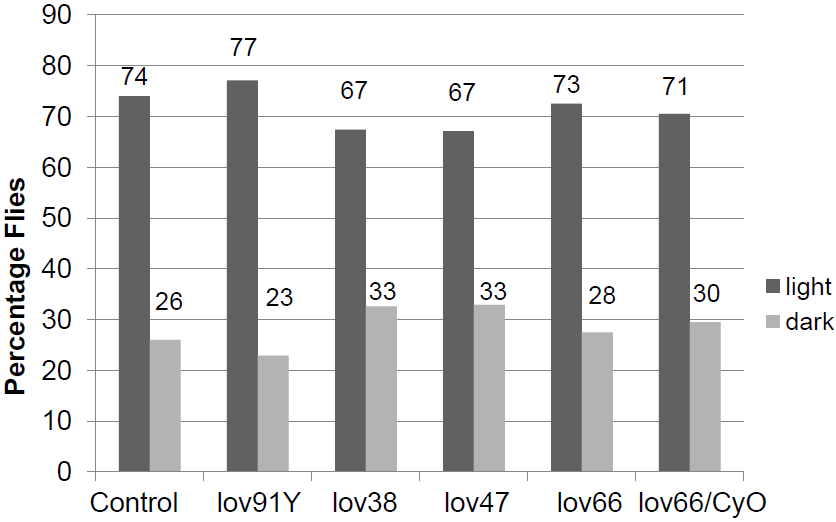


**FIGURE S2.**

Supplement: Figure S2 — lov mutants show normal responses in a fast phototaxis assay. The various lov mutants were tested for their ability to detect and respond to light. Flies were placed in a t-test apparatus in which one tube was exposed to a light source and one tube remained in the dark. The flies were allowed one minute to choose which tube to enter and then the number of flies in each tube was counted. For this assay, vials of up to 25 newly eclosed males were collected and allowed to age for three to five days before testing. The assay was repeated up to six times. Wild type flies show positive phototaxis. The responses of all lov mutants are indistinguishable from the control line (df 5; χ2 = 5.17; Control = Canton S n = 100, lov91Y n = 170, lov38 n = 132, lov47 n = 82, lov66 n = 138, lov66/CyO n = 146). (DOCX) [file pone.0061270.s002.docx]

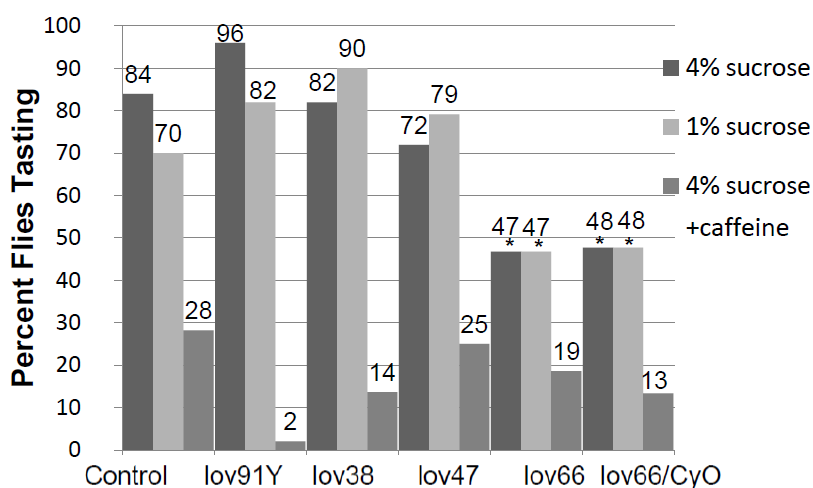


**FIGURE S3.**

Supplement: Figure S3 — lov66 has a decreased response to sucrose. The proboscis extension response (PER) assay of Gordesky-Gold (Chemical Senses 33, 301–309, 2008) was used to investigate adult taste responses. Newly eclosed males were starved overnight in food vials with paper tissue moistened with water. Flies were then mounted on toothpicks with Tissue Tack and allowed to recover for three hours. Prior to testing, flies were satiated with water to ensure that tastant, and not water, responses, were assayed. Attractive (1% or 4% sucrose) or repellent (4% caffeine +4% sucrose) tastants were touched to the front legs and the proboscis response noted. Flies were tested three times, and given water between testings. A score of one (“tasting”) was given if a fly extended its proboscis all three times. Two additional trials were performed for flies with mixed responses in the first three trials. After five trials, any fly that extended its proboscis three or more times was scored as “tasting”. Approximately 50 flies from each line were tested. A chi square test was used to determine significance relative to the control, Ore R flies. The reduced response of lov66 to sucrose and stronger response of lov91Y to sucrose+caffeine are statistically significant. (4% Sucrose: df 5; Ore R n = 50, lov91Y n = 50, lov38 n = 50, lov47 n = 50, lov66 n = 47 (χ2 = 56.7), lov66/CyO n = 44 (χ2 = 56.7); 1% Sucrose: df 5; Ore R n = 50, lov91Y n = 50, lov38 n = 50, lov47 n = 48, lov66 n = 47 (χ2 = 50.2), lov66/CyO n = 44 (χ2 = 50.2); 4% Sucrose and 4% Caffeine: df 5; Ore R n = 50, lov91Y n = 50 (χ2 = 13.5), lov38 n = 50, lov47 n = 50, lov66 n = 51, lov66/CyO n = 51). (DOCX) [file pone.0061270.s003.docx]

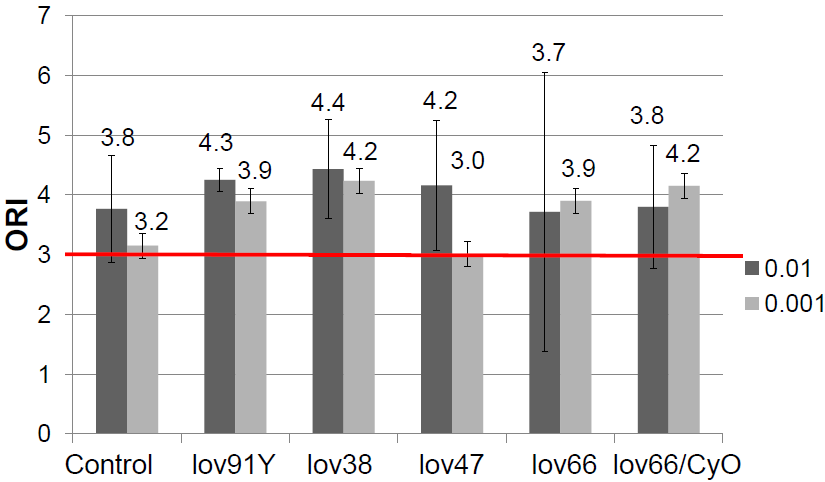


**FIGURE S4.**

Supplement: Figure S4 — Olfactory responses to a repellent odorant for lov mutants. Newly eclosed males were collected, aged for four to six days, then placed in empty food vials in groups of five. A Q-tip pre-soaked with 100 µl of repellent odorant (1% or 0.1% benzaldehyde) was then placed in the vial so that the tip was in the middle of the vial. For one minute, the number of flies at the far end of the vial (opposite the plug) was counted every five seconds. The number of flies at the far end was averaged to give an Olfactory Response Index (ORI). The assay was repeated ten times for each genotype. An ORI above 3 (above the red line) means flies are repelled by the odorant. A one-way ANOVA was performed to determine if there were differences among the lines. A Dunnett’s Test was used to compare the results of the mutant lines to the control, Ore R. Error bars represent standard error. Responses of the lov mutants to 1% benzaldehyde are indistinguishable from the control, Ore R, response (1% benzaldehyde: p = 0.593, n = 50 for all genotypes). The slightly stronger responses of lov91Y, lov38, lov66 and lov66/CyO to 0.1% benzaldehyde as compared to the control are statistically significant. (0.1% benzaldehyde: p = 0.004; n = 50 for all genotypes). Assay modified from Anholt and Mackay, Behav. Genet. 31,17–27, 2001. (DOCX) [file pone.0061270.s004.docx]

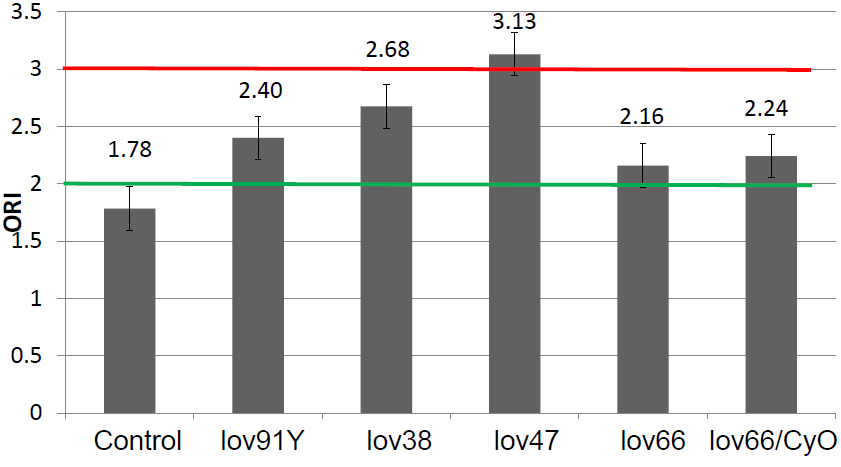


**FIGURE S5.**

Supplement: Figure S5 — Olfactory responses to a neutral odorant for lov mutants. Newly eclosed males were collected, aged four to six days and then placed in an empty food vials in groups of five. A Q-tip pre-soaked with 100 µl of neutral odorant (water) was then placed in the vial so that the tip was in the middle of the vial. For one minute, the number of flies at the far end of the vial (opposite the plug) was counted every five seconds. The number of flies at the far end was averaged to give an Olfactory Response Index (ORI). The assay was repeated at least ten times so that a minimum of 50 flies were tested. An ORI between 2 and 3 (between the green and red lines) means flies are neutral to the odorant. A one-way ANOVA was performed to determine if there were differences among the lines. A Dunnett’s Test was used to compare the results of the mutant lines to the control, Canton S. Error bars represent standard error. The slight attraction to water shown by the control and the slight repulsion to water shown by lov47 are statistically significant. (p≤0.001; Control = Canton S n = 125, lov91Y n = 50, lov38 n = 50, lov47 n = 100, lov66 n = 50, lov66/CyO n = 50). Assay modified from Anholt and Mackay, Behav. Genet. 31,17–27, 2001. (DOCX) [file pone.0061270.s005.docx]

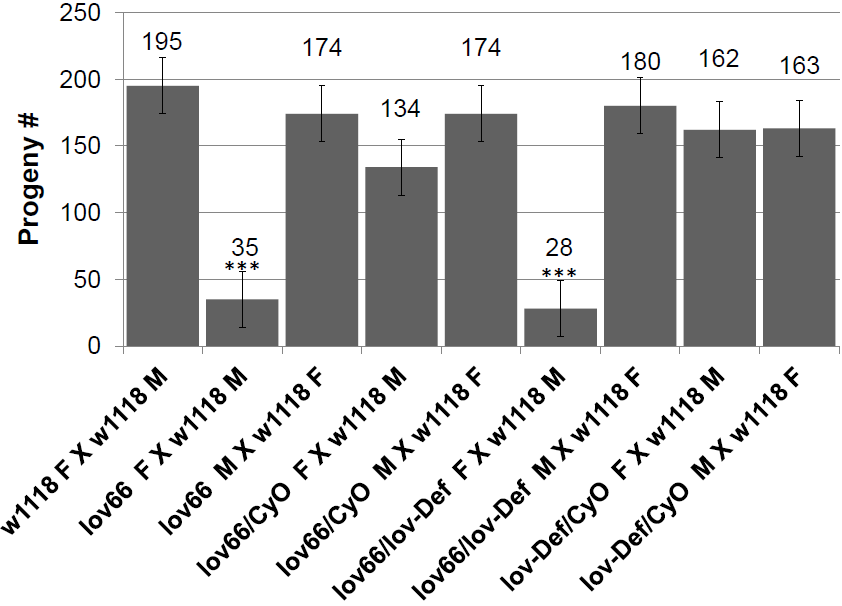


**FIGURE S6.**

Supplement: Figure S6 — lov66 decreased fertility is attributed to the females. Average total progeny number for the crosses shown above was determined as follows. Single male/single virgin female mating pairs were set up in vials and transferred on day 8 and then day 16 to new vials. On day 24 they were removed from the third vial and discarded. Adult offspring were then collected from each vial for eight days after eclosion of the first adult. A minimum of 10 mating pairs were scored for each line. Female lov66 mutants have significantly decreased fertility when compared to lov66 males in terms of producing viable progeny when mated to control flies. A one-way ANOVA was performed to determined to assess significance of differences between crosses. A Dunnett’s Test was performed with w1118 as the control. Error bars represent standard error. *** = p<0.001 relative to the w1118 control; M = males; F = females. (DOCX) [file pone.0061270.s006.docx]

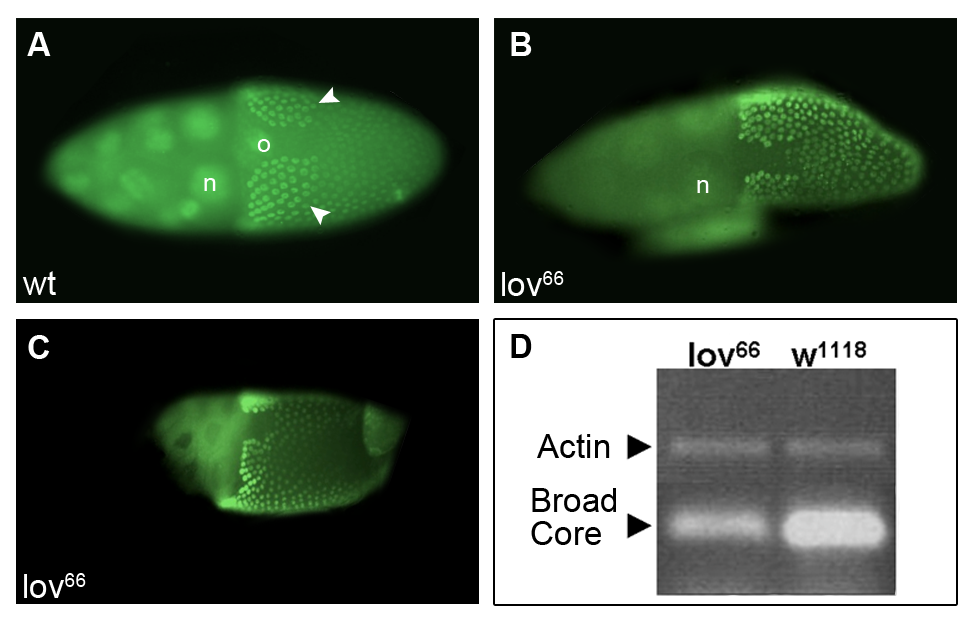


**FIGURE S7.**

Supplement: Figure S7 — lov66 affects Broad expression in the ovary. In stage 10 of oogenesis, two patches of somatic follicle cells on the dorsal surface of the oocyte compartment show elevated nuclear expression of Broad (Tzolovsky et al., Genetics 153,1371–1383, 1999). This enhanced Broad expression commits the affected cells to dorsal appendage formation. A. The Broad patches (arrowheads) on a stage 10 wild type Canton-S egg chamber. Broad staining of the nurse cell nuclei (n) and oocyte nucleus (o) is artifactual and was intermittently seen with the antibody used. B. A normal size stage 10 egg chamber from a lov66 mother. Although the overall pattern of Broad expression is similar to wild type, enhanced Broad expression at the position of the two presumptive patches of dorsal appendage cells is barely detectable. C. A short stage 10 egg chamber from a lov66 mother. Again, overall patterning of Broad expression is relatively normal but expression within the patches is highly aberrant. D. Semi-Q RT-PCR to probe for all broad transcripts within ovaries from one-day-old control (w1118) and lov66 homozygous females shows that Broad expression is depressed in lov66 ovaries. Methods. A-C. Ovaries from Canton-S or lov66 homozygous mothers were fixed in 4% paraformaldehyde and stained with the Broad-core monoclonal antibody 25E9-D7 (1∶250 dilution) from the Developmental Studies Hybridoma Bank and an Alexafluor-488 labelled goat anti-mouse secondary antibody (Invitrogen,1∶500 dilution). The Broad-core antibody recognizes sequences common to all isoforms of Broad. D. Semi-Q RT-PCR on ovarian RNA was performed as described in Material and Methods of the main text. Primers termed Broad F1 (5′ TGCAGGATGTCAACTTCATGGACC 3′) and Broad R (5′TATCTGAGCCAGATGGCTGTGTGT 3′), which span an exon-exon junction within the shared protein coding sequences of all broad transcripts, were used to probe for all processed broad transcripts. Actin 57B primers (see main text Material and Methods) were used in parallel [file pone.0061270.s007.docx]
