# Supplementary material for: The Drosophila BTB Domain Protein Jim Lovell Has Roles in Multiple Larval and Adult Behaviors
Source: PLoS One. 2013 Apr 19;8(4):e61270. doi: 10.1371/journal.pone.0061270 (PMC3631165; doi:10.1371/journal.pone.0061270)
Supplement: Table S1 — Sperm transfer and storage is not impaired in lov66 mutants. A third chromosome don juan-GFP construct (Santel et al. Mech. Dev. 64, 19–30, 1997), which generates GFP-expressing sperm, was used to monitor sperm storage in the seminal vesicles and spermathecae of mated females. Males and virgin females were aged for 3–6 days after eclosion and set up in matings of the genotypes indicated. The sperm storage organs of females were dissected out after confirmed mating and were examined for GFP fluorescing sperm. There is no significant difference in the ability of lov66 males to transfer sperm or lov66 females to store sperm, relative to the w1118 and lov66/CyO controls. χ2 = 1.04. (DOCX) [file pone.0061270.s008.docx]

| Male partner | *dj8-GFP* | *dj8-GFP* | *dj8-GFP* | *lov^66^* ;  *dj8-GFP* | *lov^66^* ;  *dj8-GFP* |
| --- | --- | --- | --- | --- | --- |
| Female partner | *w^1118^* | *lov^66^*/*CyO* | *lov^66^* | *lov^66^* /*CyO* | *lov^66^* |
| # sperm storage  organs examined | 13 | 18 | 15 | 13 | 15 |
| # sperm storage organs with GFP sperm | 10 | 12 | 12 | 9 | 10 |
| % females storing sperm | 76.9 | 66.7 | 80.0 | 69.2 | 66.7 |

**TABLE S1**
